# IgG-Independent Co-aggregation of FcεRI and FcγRIIB Results in LYN- and SHIP1-Dependent Tyrosine Phosphorylation of FcγRIIB in Murine Bone Marrow-Derived Mast Cells

**DOI:** 10.3389/fimmu.2018.01937

**Published:** 2018-08-27

**Authors:** Mathias Gast, Christian Preisinger, Falk Nimmerjahn, Michael Huber

**Affiliations:** ^1^Medical Faculty, Institute of Biochemistry and Molecular Immunology, RWTH Aachen University, Aachen, Germany; ^2^Proteomics Facility, IZKF, RWTH Aachen University, Aachen, Germany; ^3^Institute of Genetics at the Department of Biology, Friedrich-Alexander-University Erlangen-Nürnberg, Erlangen, Germany

**Keywords:** dose-response, Fc receptor, phospho-proteomics, submembranous cytoskeleton, SHIP1 recruitment

## Abstract

Activation of the high-affinity receptor for IgE (FcεRI) follows a bell-shaped dose-response curve. Upon supra-optimal stimulation, mast cell effector responses are down-regulated by inhibitory molecules like the SH2-containing inositol-5′-phosphatase SHIP1 and the SRC-family-kinase LYN. To identify further molecules involved in a negative regulatory signalosome, we screened for proteins showing the same pattern of tyrosine phosphorylation as SHIP1, which is tyrosine-phosphorylated strongest upon supra-optimal antigen (Ag) stimulation. The low-affinity IgG receptor, FcγRIIB, was found to be most strongly phosphorylated under supra-optimal conditions. This phosphorylation is the consequence of passive, Ag/IgE-dependent and progressive co-localization of FcεRI and FcγRIIB, which is not dependent on IgG. Upon supra-optimal FcεRI cross-linking, FcγRIIB phosphorylation is executed by LYN and protected from dephosphorylation by SHIP1. Analysis of FcγRIIB-deficient bone marrow-derived mast cells revealed an ambiguous phenotype upon FcεRI cross-linking. Absence of FcγRIIB significantly diminished the level of SHIP1 phosphorylation and resulted in augmented Ca^2+^ mobilization. Though, degranulation and IL-6 production were only weakly altered. Altogether our data establish the LYN/FcγRIIB/SHIP1 signalosome in the context of FcεRI activation, particularly at supra-optimal Ag concentrations. The fact that SHIP1 tyrosine phosphorylation/activation not only depends on FcγRIIB, highlights the necessity for its tight backup control.

## Introduction

The high-affinity receptor for IgE (FcεRI) is the central activating receptor of mast cells (MCs) involved in type I hypersensitivity reactions such as allergic asthma and food allergy. IgE-bound FcεRIs can be activated via cross-linking by specific multivalent Ag/allergen. Pre-formed mediators like histamine and proteases are immediately released from secretory lysosomes, metabolites of arachidonic acid are generated, and *de-novo* synthesis of pro-inflammatory cytokines is initiated.

A specific feature of the FcεRI is its unusual bell-shaped dose-response behavior. The cellular response caused by FcεRI cross-linking increases until the use of optimal doses of Ag and descends again at higher (supra-optimal) Ag concentrations. Careful studies have demonstrated that the descending part of the dose-response curve is actively controlled by inhibitory molecules and cellular mechanisms (1).

One of these mechanisms is the cytoskeletal rearrangement caused by FcεRI-mediated MC activation. Ag stimulation not only triggers formation of FcεRI complexes, but also their detergent-resistant association to the submembranous cytoskeleton (SMC) (2). With increasing Ag concentrations and concomitant increase in the degree of receptor cross-linking, association of FcεRI to the cytoskeleton increases, in fact beyond the Ag concentration needed for maximum activation of the MCs (2). This is accompanied by the dose-dependent polymerization of the submembranous F-actin meshwork that negatively interferes with MC activation, especially at supra-optimal Ag concentrations. Inhibition of actin polymerization increases degranulation, particularly upon supra-optimal stimulation, while at the same time detergent-insolubility of the FcεRI is decreased (3–5).

Besides cytoskeletal rearrangements also other observations indicate active control of MC activation under supra-optimal conditions. Early overall tyrosine phosphorylation was shown to be even slightly enhanced in supra-optimally compared to optimally stimulated MCs (5, 6), which contradicts the bell-shaped form of the dose-response curve. Particularly the SH2-containing inositol polyphosphate-5′-phosphatase SHIP1 displays a pattern of tyrosine phosphorylation, which increases in parallel to Ag concentration (5, 7). SHIP1, as a negative regulator of PI3K signaling (5), hydrolyses phosphatidylinositol-3,4,5-trisphosphate (PIP_3_) to PI-3,4-P_2_ (8). Deficiency of SHIP1 causes a hyperactive phenotype in bone marrow-derived MCs (BMMCs), with only weak or no reduction in degranulation after supra-optimal stimulation (5). Hence it was characterized as a central negative regulator of supra-optimal FcεRI signaling. Besides SHIP1 also the SRC family kinase LYN contributes to negative signaling in MCs by tyrosine phosphorylating and activating SHIP1 (9). Furthermore, protein kinase C-δ (PKC-δ) is part of such inhibitory signalosome by complexing with SHIP1 and LYN (10–12). Like SHIP1, LYN was found to be most strongly activated at supra-optimal Ag conditions resulting in increased SHIP1 phosphorylation as well as decreased degranulation and cytokine production (13). Taken together, several findings converge to support the idea of a tight negative control of FcεRI-triggered MC effector responses at supra-optimal Ag concentrations.

Another well-known control mechanism of FcεRI signaling is based on coaggregation of the low-affinity receptor for IgG, FcγRIIB, to FcεRI (14). This mechanism requires IgG-containing immune complexes that can cross-link FcγRIIB to IgE-loaded FcεRI or FcεRI-IgE-Ag complexes in stimulated MCs (14). The recruitment of FcγRIIB causes the attenuation of FcεRI-triggered MC activation in a mechanism also depending on LYN and SHIP1.

In this study, we show an IgG-independent mechanism of FcγRIIB recruitment that is based on Ag concentration-dependent, progressive co-localization of FcεRI and FcγRIIB. Thereby, the FcγRIIB immunoreceptor tyrosine-based inhibition motif (ITIM) is progressively phosphorylated by LYN and protected by SHIP1 in parallel to increasing Ag concentrations. In line, loss of FcγRIIB significantly decreased SHIP1 phosphorylation upon supra-optimal stimulation. Finally, our observations indicate that FcγRIIB contributes to supra-optimal negative regulation of FcεRI-induced MC activation by serving as a membrane adaptor for SHIP1.

## Results

### Tyrosine phospho-proteome analysis for the identification of proteins potentially involved in supra-optimal FcεRI signaling

**Figure 1:**
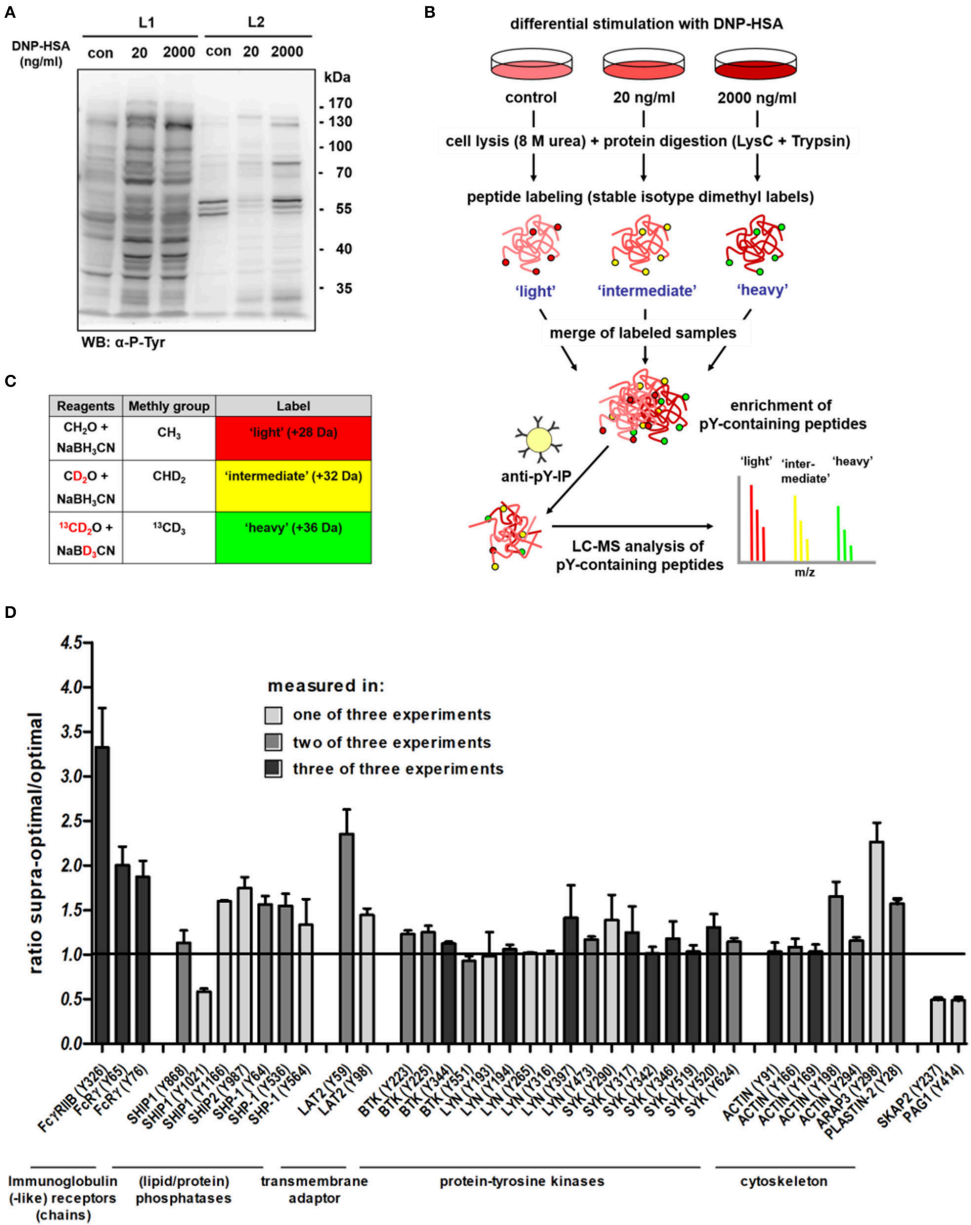
Screening for proteins that display increased tyrosine phosphorylation after supra-optimal stimulation. **(A)** WT BMMCs sensitized with DNP-specific IgEs (SPE-7; 0.15 μg/ml) were stimulated with either an optimal (20 ng/ml) or a supra-optimal (2,000 ng/ml) concentration of DNP-HSA for 5 min. Cells were consecutively lysed in two different buffers (L1: 0.5% Triton X-100 only; L2: 0.5% Triton X-100, 0.2% deoxycholate and 0.01% SDS) and lysates were subjected to SDS-PAGE and anti-P-Tyr immunoblotting. **(B)** Overview of the quantitative proteomics workflow [adapted from Boersema et al. (15)]. A detailed description of the procedure is provided in the respective Materials and Methods section Quantitative Proteomics of P-Tyr Containing Peptides. **(C)** Labeling strategy. Combinations of different isotopomers of formaldehyde and cyanoborohydride were used to create stable isotope dimethyl labels. All primary amines of the peptides were converted to dimethylamines. Mass difference of 4 Da between each of the labels allowed an immediate quantitative comparison in a single MS analysis. **(D)** Changes in tyrosine phosphorylation measured with LC-MS. Ratio of tyrosine phosphorylation in supra-optimally compared to optimally stimulated cells, selected features. All results shown in this figure are representatives of at least 3 independent experiments.

To identify further proteins potentially involved in supra-optimal negative regulation, we first addressed if other tyrosine-phosphorylated proteins besides SHIP1 and FcεRI can also interact with the actin cytoskeleton in a detergent-resistant manner upon Ag stimulation. Therefore, we sensitized MCs with the DNP- specific IgE SPE-7 overnight followed by addition of DNP-HSA at an optimal (20 ng/ml) as well as a supra-optimal (2,000 ng/ml) concentration. Differentially stimulated BMMCs were lysed first in a mild buffer (L1: 0.5% Triton X-100) and after centrifugation the resulting pellets were further lysed under more stringent conditions (L2: 0.5% Triton X-100, 0.01% SDS and 0.2% deoxycholate), solubilizing proteins still associated to the SMC (Figure 1A). After optimal stimulation (20 ng/ml), most tyrosine-phosphorylated proteins were found in the L1 lysate (Figure 1A), while upon treatment with a supra-optimal Ag concentration (2,000 ng/ml), some proteins remained in the pellet after L1 lysis and only were solubilized upon L2 treatment (Figure 1A). Provided that increased insolubility conforms to suppression of degranulation, some of these proteins might be involved in negative regulation of FcεRI-mediated MC activation.

Therefore, we sought to directly compare the patterns of overall tyrosine phosphorylation in differentially stimulated BMMCs using a stable isotope dimethyl labeling-based quantitative mass spectrometry approach (15, 16). The respective workflow is depicted in Figure 1B (adapted from (17); detailed description in the Materials and Methods section). For stable isotope dimethyl labeling, peptides were exposed to isotopomers of formaldehyde and cyanoborohydride (Figure 1C). Thus, all primary amines (N-termini and lysine side chains) were converted to dimethylamines (18). By using different combinations of the labeling reagents for each sample, three different labels were introduced and peptides with different masses (+4 Da) were generated (Figure 1C). Hence, this method allowed distinguishing between three distinct peptide pools (e.g., light = control, intermediate = optimal stimulation, and heavy = supra-optimal stimulation) after combination of the three labeled samples in a single mass spectrometry analysis. Tyrosine-phosphorylated peptides were enriched by immunoprecipitation and analyzed by liquid chromatography–mass spectrometry (LC-MS) (15).

Dose-dependent changes in overall tyrosine phosphorylation after differential Ag treatment were quantified. Several proteins were identified that displayed increased phosphorylation at one or more tyrosine residues after supra-optimal compared to optimal stimulation. A selection of these proteins is shown in Figure 1D. A complete list with proteins identified in the quantitative MS approach is provided in Supplementary Tables 1, 2. Concerning the molecules depicted in Figure 1D, the biggest increase in tyrosine phosphorylation was observed for the low-affinity receptor for IgG, FcγRIIB (CD32B) with phosphorylation at Y326 showing a 3.3-fold increase in supra-optimally stimulated cells, over the optimally stimulated ones. Only one protein, which was Ubash3b, displayed an even stronger increase in tyrosine phosphorylation after supra-optimal stimulation (Supplementary Tables 1, 2). As this protein was only identified once in our MS experiments, we did not further analyze it. FcγRIIB was the most interesting target for further investigations, as we were able to reproducibly identify it and as it is known to strongly interact with SHIP1, which is a major negative regulator in supra-optimal FcεRI signaling (5, 7). Besides phosphorylation of Y326, also phosphopeptides containing Y309 in the ITIM sequence of FcγRIIB, which directly binds the SH2-domain of SHIP1 (19), were also measured, though less frequently. Other proteins also showed increased tyrosine phosphorylation after supra-optimal stimulation, albeit less pronounced. Though this is only a selection of proteins, the experiments clearly showed that overall tyrosine phosphorylation in general does not match with the bell-shaped dose-response curve (see Supplementary Tables 1, 2). Phosphorylation of various proteins seemed to be on a similar level or even slightly increased upon supra-optimal vs. optimal stimulation. The only two molecules that displayed a significant down regulation upon supra-optimal stimulation when compared to optimal stimulation were SKAP2 (Y237) and PAG1 (Y414) (Figure 1D; Supplementary Tables 1, 2). This underlines the conclusions drawn from previous studies proposing a negative regulatory signalosome that actively down-regulates FcεRI signaling upon stimulation with supra-optimal Ag concentrations.

### FcγRIIB is tyrosine-phosphorylated upon supra-optimal ag stimulation in a passive and IgG-independent manner

**Figure 2:**
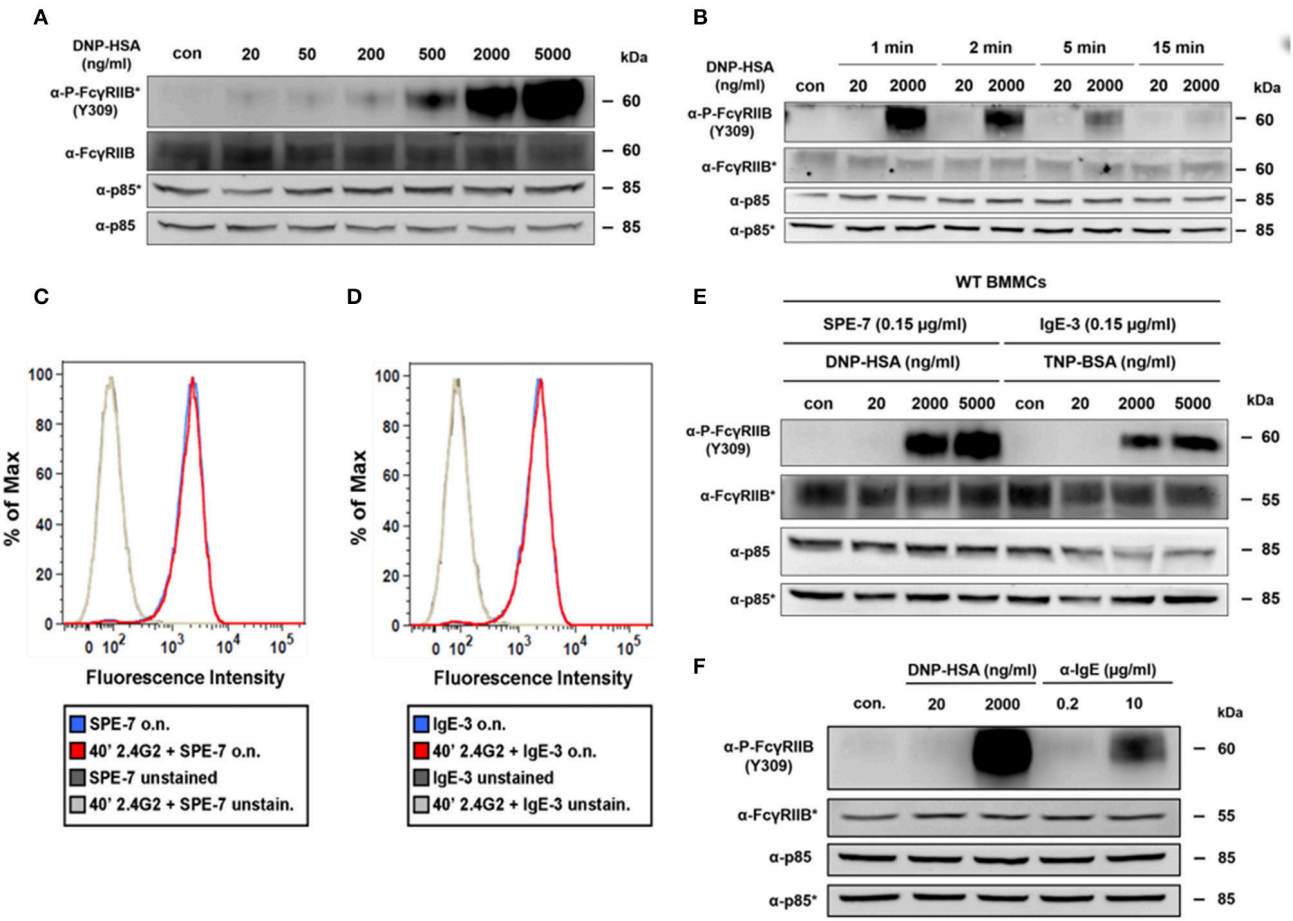
Passive, IgG-independent phosphorylation of the FcγRIIB ITIM domain at supra-optimal Ag concentrations. **(A)** IgE-sensitized BMMCs (0.15 μg/ml SPE-7) were stimulated for 2 min with increasing concentrations of DNP-HSA as depicted. Lysed cells were subjected to SDS-PAGE and subsequently to P-FcγRIIB and FcγRIIB-specific immunoblotting (loading control: anti-p85). Pictures of the full blots are shown in Supplementary Figure 12. (**B)** IgE-sensitized BMMCs (0.15 μg/ml SPE-7) were stimulated as depicted for 2 min and after lysis subjected to SDS-PAGE and immunoblotting. Pictures of the full blots are shown in Supplementary Figure 13. **(C)** BMMCs were treated with 2.4G2 for 40 min to block Fc-binding sites of FcγRIIB/FcγRIII or were left untreated instead. Both samples were then sensitized with SPE-7 overnight (o.n.) and the amount of IgE on the cell surface was determined by flow cytometry. **(D)** The experiment shown in Figure 2C was repeated using IgE-3 instead of SPE-7. **(E)** WT BMMCs were either sensitized with SPE-7 (0.15 μg/ml) or a respective amount of IgE-3 and stimulated as indicated with increasing concentrations of specific Ags (DNP-HSA for SPE-7 and TNP-BSA for IgE-3) for 2 min. SDS-PAGE and immunoblotting were used to show FcγRIIB phosphorylation. Pictures of the full blots are shown in Supplementary Figure 14. **(F)** WT BMMCs sensitized with SPE-7 (0.15 μg/ml) were stimulated with the indicated concentrations of DNP-HSA or α-IgE respectively for 2 min. Pictures of the full blots are shown in Supplementary Figure 15. **(A,B,E,F)** As proteins of comparable size were analyzed, lysates were separated on two gels, which were blotted on two membranes with an anti-p85 loading control on each membrane (detections belonging to one membrane marked with/without asterisk). All results shown in this figure are representatives of at least 3 independent experiments.

The strong increase in tyrosine phosphorylation of FcγRIIB at high Ag concentrations was verified by phospho-specific Western blotting (Figures 2A,B). While almost no phosphorylation of the FcγRIIB ITIM was detectable at optimal Ag concentrations (20 ng/ml), it strongly increased dose-dependently between 200 and 5,000 ng/ml (Figure 2A). This pattern, not matching the bell-shaped dose-response curve, was comparable to the described pattern of SHIP1 tyrosine phosphorylation (5). Furthermore, Ag-dependent FcγRIIB phosphorylation was rather transient (Figure 2B). While the signal was strong after 1 min of stimulation, it quickly decreased, and almost ceased completely after 15 min (Figure 2B).

Via IgG-containing immune complexes, FcγRIIB is cross-linked to activating Fc receptors on various cell types, as well as the BCR on B-cells (14, 20). On MCs, such co-ligation to FcγRIIB negatively interferes with FcεRI-mediated activation through the recruitment of SHIP1 (14, 21). It is important to note that the setup in the present study did neither contain IgG nor IgG-containing immune complexes. Hence, we assumed that the observed FcγRIIB phosphorylation happened in an IgG-independent manner. Kinet and colleagues previously demonstrated that FcγRIII (CD16) and FcγRII (CD32) can also function as low-affinity receptors for IgE (22). Since in our present study BMMCs were first loaded overnight with a rather low dose of IgE (0.15 μg/ml) and then thoroughly washed before subsequent Ag stimulation, the formation of IgE-containing immune complexes might most likely be excluded as trigger for FcγRIIB activation.

Additionally, high-affinity binding of free IgE to FcγRIIB under our experimental conditions was excluded. Blocking of FcγRIIB with the CD32/CD16-specific mAb 2.4G2 (23) prior to sensitization with IgE did not affect the amount of IgE on the cell surface compared to BMMCs sensitized with IgE in the absence of 2.4G2 (Figure 2C).

Furthermore, also the cytokinergic properties of the used IgE (SPE-7) (24) are not related to FcγRIIB activation observed in this study. Bax et al. showed that high concentrations of SPE-7 (~2–3 μg/ml) are needed to cause these cytokinergic effects (25), which are especially driven by dissociation-resistant SPE-7 trimers (26). Despite only low concentrations of SPE-7 (0.15 μg/ml) were used in this study, we repeated the experiment described in Figure 2C with a non-cytokinergic IgE (IgE-3) instead of SPE-7 (24). Again, fluorescence intensity was not affected by blocking CD16/32 with 2.4G2 (Figure 2D). Additionally, the FcγRIIB tyrosine phosphorylation pattern in response to Ag stimulation did not differ, when BMMCs were sensitized with IgE-3 (0.15 μg/ml) instead of SPE-7 (Figure 2E).

To also consider the specific multivalent nature of the DNP-HSA that was initially used as an Ag to trigger the observed FcγRIIB phosphorylation, we also tested whether only bivalent cross-linking of the FcεRI could cause this pattern of FcγRIIB phosphorylation. For that purpose, FcεRI cross-linking was either induced with an optimal and a supra-optimal amount of DNP-HSA or optimal and supra-optimal concentrations of monoclonal anti-IgE respectively (Figure 2F). Comparison of those stimuli showed that in both cases optimal stimulation was not able to trigger FcγRIIB phosphorylation whereas supra-optimal stimulation distinctly did (Figure 2F). It must be noted that stimulation with anti-IgE does not perfectly resemble stimulation with a linear bivalent Ag. Further, interaction between FcγRIIB and the anti-IgE (IgG_1_κ) used for this experiment is potentially possible, meaning that recruitment of FcγRIIB would not be IgG-independent in the given experimental setup. Nevertheless, strong interaction between anti-IgE and FcγRIIB is unlikely, as the respective antibody was rat-derived, and MCs were of murine origin. Previous studies suggest that compared to murine IgG_1_, rat IgG_1_ only weakly interacts with the murine FcγRIIB (27, 28). Particularly in the publication by Siragam et al., a monoclonal rat anti-integrin α_IIb_ antibody (isotype IgG_1_κ) was used to induce thrombocytopenia in mice, which showed no difference between WT and *Fcgr2*^−/−^ mice.

In conclusion, FcγRIIB tyrosine phosphorylation in IgE-loaded BMMCs in response to stimulation with supra-optimal Ag concentrations occurred in an IgG-independent and thus passive fashion.

### Passive activation of FcγRIIB at supra-optimal Ag concentrations depends on LYN as well as on SHIP1, and affects SHIP1 recruitment

**Figure 3:**
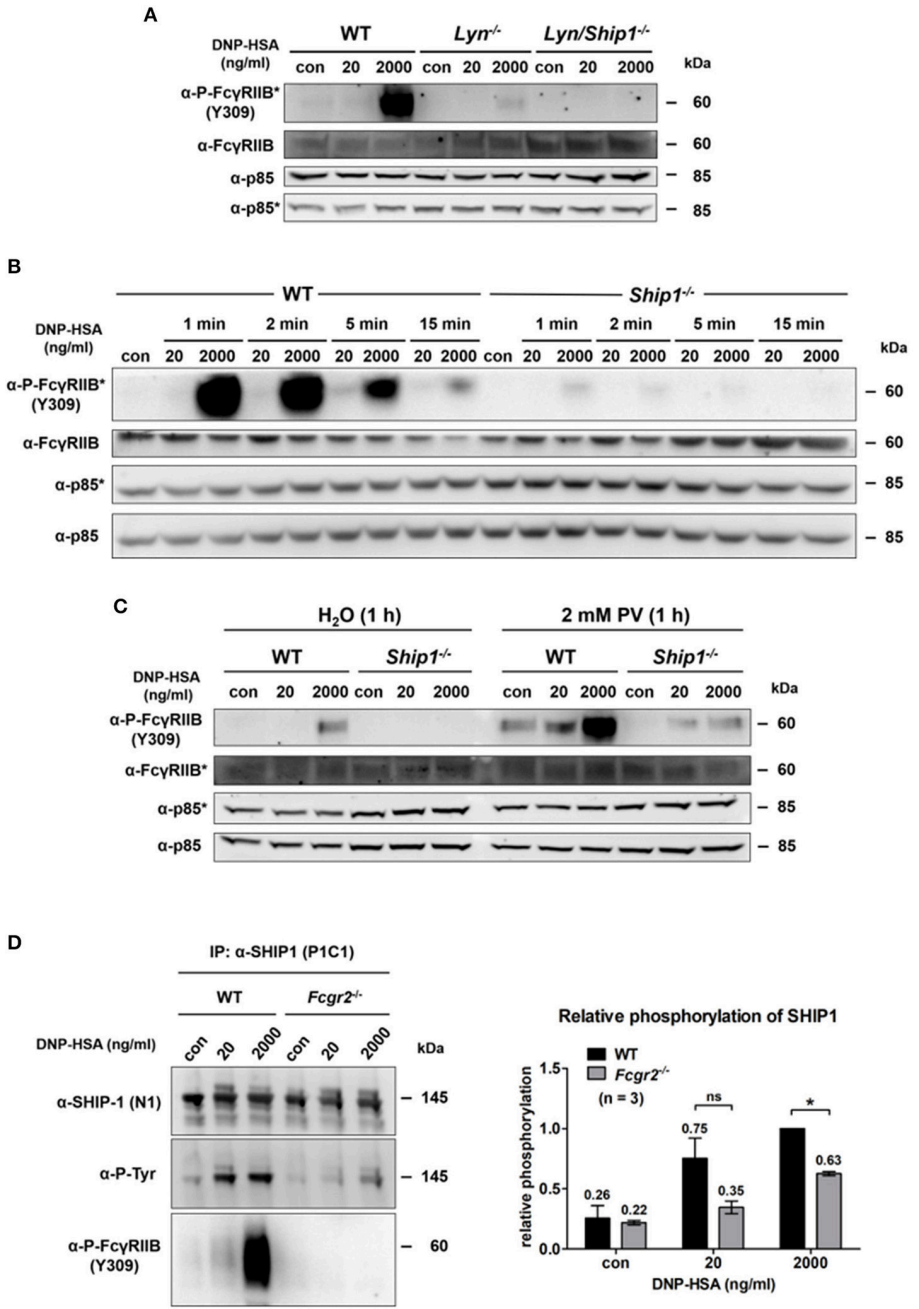
Passive activation of FcγRIIB depends on Lyn as well as SHIP1 and contributes to SHIP1 membrane recruitment. **(A)** Murine WT, *Lyn*^−/−^ and *Lyn/ Ship1*^−/−^ BMMCs sensitized with SPE-7 (0.15 μg/ml), were challenged as indicated for 2 min. Cells were lysed and subjected to SDS-PAGE and subsequently to P-FcγRIIB and FcγRIIB-specific Western blotting (loading control: anti-p85). Pictures of the full blots are shown in Supplementary Figure 16. **(B)** WT and *Ship1*^−/−^ BMMCs sensitized with SPE-7 (0.15 μg/ml) were stimulated as indicated. Cells were lysed and subjected to SDS-PAGE and Western blotting. Pictures of the full blots are shown in Supplementary Figure 17. **(C)** WT and *Ship1*^−/−^ BMMCs were sensitized with SPE-7 (0.15 μg/ml). Cells were treated with 2 mM of the phosphatase inhibitor PV or the respective amount of the solvent H_2_O for 1 h prior to 2 min of Ag stimulation with the depicted concentrations of DNP-HSA. Cells were lysed and subjected to SDS-PAGE and Western blotting. Pictures of the full blots are shown in Supplementary Figure 18. **(A–C)** As proteins of comparable size were analyzed, lysates were separated on two gels, which were blotted on two membranes with an anti-p85 loading control on each membrane (detections belonging to one membrane marked with/without asterisk). **(D)** SPE-7-sensitized (0.15 μg/ml) WT and *Fcgr2*^−/−^ BMMCs were challenged as indicated. MCs were lysed and immunoprecipitation against SHIP1 was conducted. SHIP1 tyrosine phosphorylation was significantly decreased in *Fcgr2*^−/−^ BMMCs (*p* = 0.0252). The bar graph shows the relative phosphorylation of SHIP1 normalized to the level of SHIP1 phosphorylation after 2,000 ng/ml DNP-HSA in WT BMMCs. Pictures of the full blots are shown in Supplementary Figure 19. Results shown in this figure are representatives of at least 3 independent experiments and in the diagram depicted as mean ± SD.

The tyrosine kinase LYN was described to phosphorylate the ITIM upon IgG-based FcγRIIB recruitment (29). Using *Lyn*^−/−^ BMMCs, we could show that LYN is responsible for the FcγRIIB phosphorylation at Y309 within the ITIM upon supra-optimal FcεRI stimulation (Figure 3A). Compared to WT cells, phosphorylation of FcγRIIB was strongly decreased in *Lyn*^−/−^ BMMCs after supra-optimal challenge. Moreover, analysis of *Lyn/Ship1* double-deficient BMMCs revealed that the additional loss of SHIP1 further decreased the level of FcγRIIB phosphorylation (Figure 3A). Thus, these data suggested that SHIP1 is not only recruited upon phosphorylation of the FcγRIIB ITIM, but it also affects ITIM phosphorylation itself. Indeed, a comparison of WT and *Ship1*^−/−^ BMMCs, stimulated with optimal and supra-optimal Ag concentrations for increasing times, revealed marked loss of FcγRIIB Y309 phosphorylation in the absence of SHIP1, despite the presence of LYN (Figure 3B). By its high-affinity binding to the tyrosine-phosphorylated ITIM, the SH2-domain of SHIP1 might protect the phospho-ITIM from dephosphorylation by a respective tyrosine phosphatase. This observation of lost FcγRIIB ITIM phosphorylation in *Ship1*^−/−^ BMMCs does of course not allow to draw any conclusion about Y326 phosphorylation, which was primarily detected in our MS experiments (Figure 1D). When phosphorylated, Y326 mediates the interaction between FcγRIIB and the SH2-domain of the adaptor protein GRB2. The C-terminal SH3-domain of GRB2 can then bind to SHIP1, which stabilizes the interaction between FcγRIIB and SHIP1 (30).

Pharmacological inhibition of tyrosine phosphatases with pervanadate (PV) for 1 h prior to Ag stimulation rescued ITIM phosphorylation of FcγRIIB in *Ship1*^−/−^ BMMCs (Figure 3C). Without phosphatase inhibition, no FcγRIIB phosphorylation was detected in supra-optimally stimulated *Ship1*^−/−^ MCs. In contrast, FcγRIIB was distinctly phosphorylated in these cells upon phosphatase inhibition. The level of tyrosine phosphorylation in *Ship1*^−/−^ BMMCs after PV treatment was similar to the phosphorylation in the control (H_2_O)-treated WT BMMCs after supra-optimal stimulation. Interestingly, upon phosphatase inhibition FcγRIIB phosphorylation was also observed in response to optimal Ag concentration (Figure 3C). This showed that in the absence of SHIP1, the FcγRIIB ITIM was in fact phosphorylated, however unprotected against dephosphorylation. Furthermore, FcγRIIB phosphorylation was strongly enhanced in unstimulated as well as optimally and supra-optimally stimulated WT BMMCs after PV treatment (Figure 3C). This indicated basal activation of FcγRIIB ITIM-specific phosphatase(s) in WT BMMCs.

Beyond, immunoprecipitation of SHIP1 from differentially stimulated WT and *Fcgr2*^−/−^ BMMCs and subsequent phospho-tyrosine-specific immunoblotting revealed that SHIP1 tyrosine phosphorylation was decreased in *Fcgr2*^−/−^ compared to WT BMMCs in response to both optimal as well as supra-optimal stimulation. BMMCs lacking FcγRIIB were generated from *Fcgr2*^−/−^ mice. These MCs showed a comparable differentiation process and expressed equal amounts of FcεRI and KIT as the respective WT (Supplementary Figure 1). A negative control for this IP was conducted in *Ship1*^−/−^ BMMCs and proved the specificity of the antibody that was used in this setup to precipitate SHIP1 (Supplementary Figure 2). After supra-optimal stimulation, SHIP1 phosphorylation was significantly decreased by 37% (*p* = 0.025) in *Fcgr2*^−/−^ (Figure 3D; right panel). Moreover, in WT BMMCs phosphorylated FcγRIIB was co-precipitated with SHIP1 not only after supra-optimal stimulation, but to a lesser extent also after optimal stimulation and even in unstimulated MCs (Figure 3D). Thus, basal FcγRIIB ITIM phosphorylation and the resulting interaction with SHIP1 appears to take place. The interaction of both proteins was markedly increased upon Ag stimulation, especially at supra-optimal concentrations. In parallel, SHIP1 phosphorylation proceeded. In conclusion, upon supra-optimal FcεRI stimulation, the FcγRIIB ITIM is phosphorylated in an immunoglobulin-independent manner by LYN, enabling the FcγRIIB to act as a membrane anchor for SHIP1.

### Augmented IgG-independent co-localization of FcγRIIB and FcεRI in response to increasing Ag concentrations

**Figure 4:**
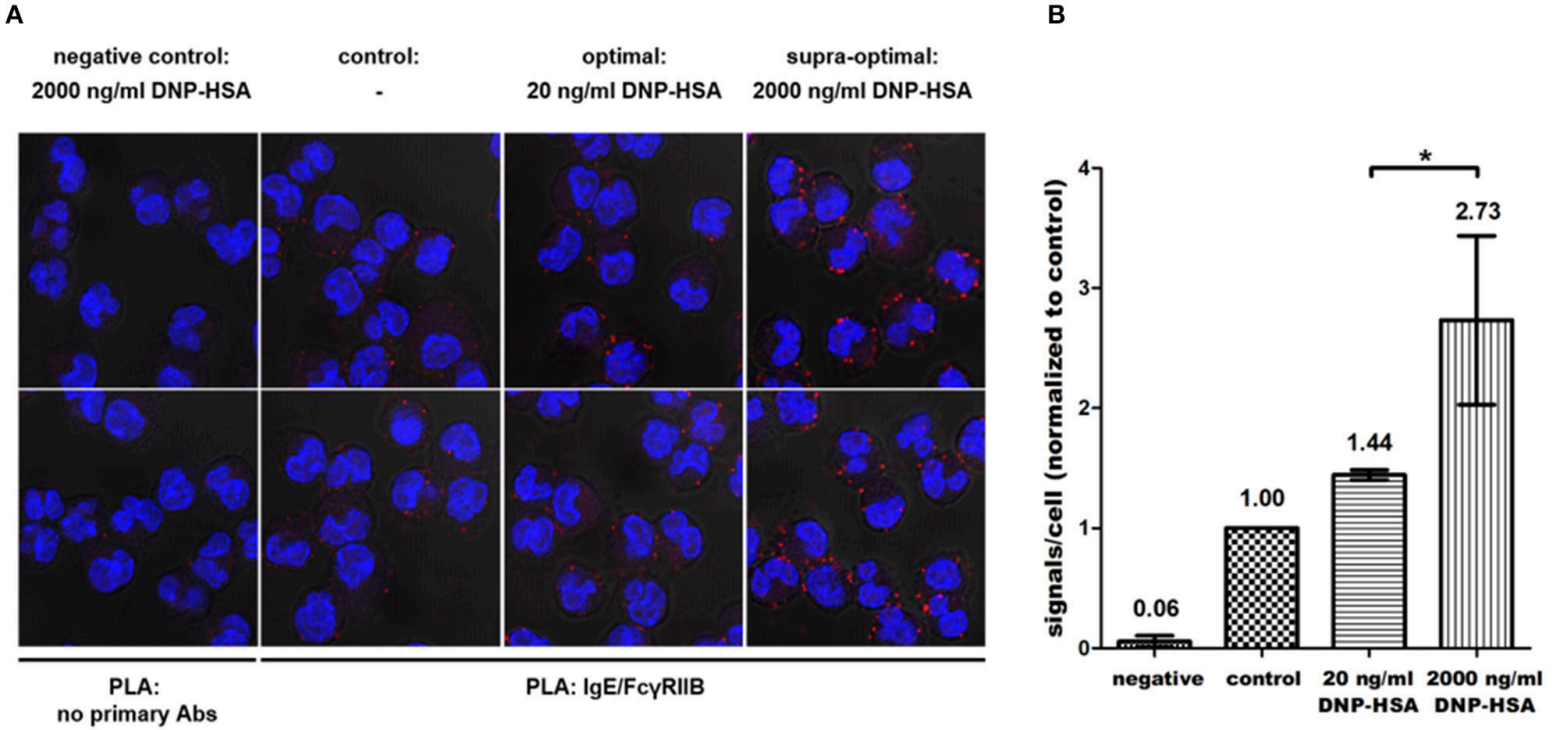
Progressive co-localization of FcγRIIB and FcεRI with increasing Ag concentrations happens in an IgG-independent manner. **(A)** *In situ* PLA was conducted with primary antibodies against murine IgE and FcγRIIB according to the protocol described in the Materials and Methods section. As a negative control, cells were incubated with the solvent of the respective primary antibodies only (left column). **(B)** For every condition 12 Z-Stack series (4 per experiment) of randomly selected areas sized 67.3 × 67.3 μm were recorded (35–50 cells each). Average amounts of fluorescence signals/cell were normalized to unstimulated controls, which was defined as 1. Co-localization was significantly increased after supra-optimal stimulation (*p* = 0.034). Results shown in this figure are representatives of 3 independent experiments and in the diagram depicted as mean ± SD. *p < 0.05.

Next, we tested whether FcγRIIB phosphorylation correlates with increased proximity of FcγRIIB and FcεRI induced by supra-optimal Ag stimulation. Given this, these receptors should show increased co-localization in parallel to increasing Ag concentrations. To measure co-localization, an *in situ* proximity ligation assay (PLA) was performed using IgE- and FcγRIIB-specific antibodies. As shown in Figure 4, co-localization of the two receptors increased with rising Ag concentrations. The negative control (left column) displayed hardly any unspecific binding of the PLA probes. This was further verified by an additional negative control using *Fcgr2*^−/−^ instead of WT BMMCs (Supplementary Figure 3). In contrast to WT-based negative controls these BMMCs were incubated with both primary antibodies. Thus, positive PLA results obtained in the actual samples were based on a close proximity of the two receptors recognized by specific primary antibodies (Figure 4A). Compared to unstimulated cells (2nd column from left), that already showed rare events of proximity between FcγRIIB and FcεRI, the number of fluorescence signals was 1.44-fold increased after optimal stimulation (*n* = 3). After supra-optimal stimulation, fluorescence signals were further increased to 2.73-fold compared to the unstimulated control (Figure 4B). These findings clearly showed that IgG-independent FcγRIIB phosphorylation at supra-optimal Ag concentrations went in line with increased proximity between FcγRIIB and FcεRI.

### Stimulus-dependent F-actin formation and unimpeded redistribution of FcγRIIB mediate ITIM phosphorylation at supra-optimal Ag concentrations

**Figure 5:**
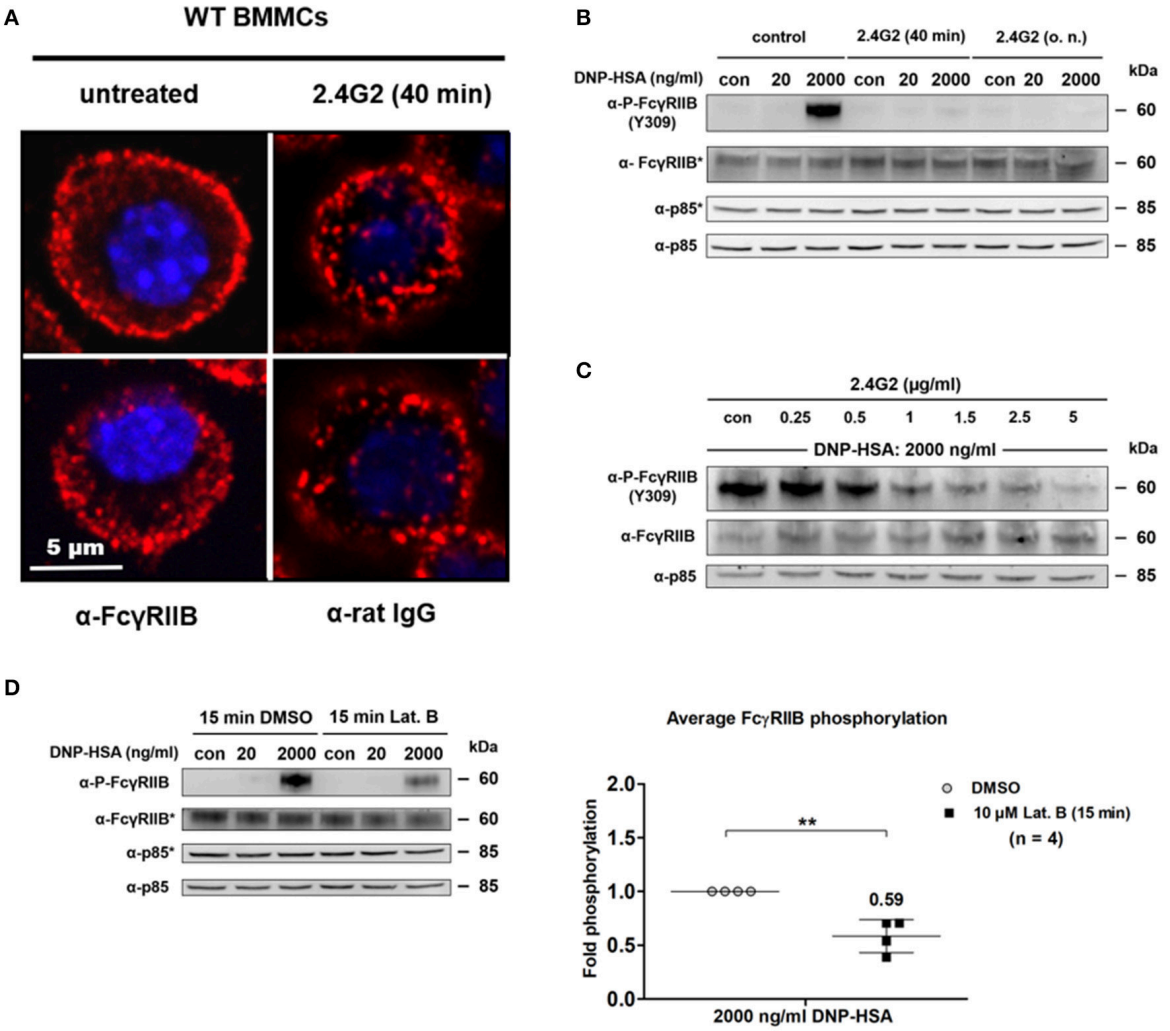
Unimpeded redistribution of FcγRIIB and stimulus-dependent F-actin formation affect ITIM phosphorylation at supra-optimal Ag concentrations. **(A)** WT BMMCs were blocked with 8 μg/ml of 2.4G2 for 40 min or left untreated instead. Cells were fixed and stained for FcγRIIB or 2.4G2 respectively. Confocal laser scanning microscopy revealed that 2.4G2 seems to cause condensation of FcγRIIB/FcγRIII. Two representative cells are shown for each condition. **(B)** WT BMMCs were either sensitized with SPE-7 (0.15 μg/ml) overnight and then treated with 2.4G2 for 40 min before stimulation or treated with 8 μg/ml of 2.4G2 for 40 min and subsequently sensitized with SPE-7 (0.15 μg/ml) overnight. Control MCs were sensitized with SPE-7 (0.15 μg/ml) only. Cells were stimulated with the indicated amounts of Ag for 2 min, lysed and subjected to SDS-PAGE and subsequently to P-FcγRIIB and FcγRIIB-specific immunoblotting (loading control: anti-p85). Pictures of the full blots are shown in Supplementary Figure 20. **(C)** SPE-7 sensitized WT BMMCs were treated with the indicated concentrations of 2.4G2 for 40 min. BMMCs were challenged with DNP-HSA (2,000 ng/ml) for 2 min, lysed and subjected to SDS-PAGE and immunoblotting. Pictures of the full blots are shown in Supplementary Figure 21. **(D)** WT BMMCs sensitized with IgE (0.15 μg/ml) were treated with 10 μM of Latrunculin B (Lat. B) or a respective amount of DMSO for 15 min. Cells were challenged as indicated for 2 min, lysed and subjected to SDS-PAGE and immunoblotting. The diagram shows average FcγRIIB phosphorylation after Lat. B treatment normalized to the level of respective phosphorylation in the DMSO controls after 2000 ng/ml DNP-HSA (*n* = 4; depicted as mean ± SD). Upon Lat. B treatment FcγRIIB phosphorylation is significantly decreased (*p* = 0.0046). Pictures of the full blots are shown in Supplementary Figure 22. **(B,D)** As proteins of comparable size were analyzed, lysates were separated on two gels, which were blotted on two membranes with an anti-p85 loading control on each membrane (detections belonging to one membrane marked with/without asterisk). The results shown in this figure are representatives of at least 3 independent experiments and in the diagram depicted as mean ± SD.

Passive FcγRIIB phosphorylation was linked to Ag-driven co-localization of increasing FcεRI clusters and FcγRIIB. We assumed that aggregation of FcγRIIB by 2.4G2 prior to Ag-induced MC stimulation could decrease FcγRIIB mobility and affect its association with FcεRI clusters as well as consequent FcγRIIB phosphorylation. We first compared the distribution of FcγRIIB on the surface of MCs that were either treated with 8 μg/ml of 2.4G2 for 40' or left untreated (Figure 5A). 2.4G2 seemed to cause aggregation of FcγRIIB/FcγRIII. While untreated cells displayed a rather consistent distribution of FcγRIIB (Figure 5A; left column), 2.4G2 treatment condensed FcγRIIB/FcγRIII to larger aggregates/clusters (right column). Moreover, aggregation of FcγRIIB by 2.4G2 also resulted in a loss of ITIM phosphorylation upon supra-optimal stimulation (Figure 5B), albeit not in increased β-hexosaminidase release (Supplementary Figure 4) and IL-6 production (Supplementary Figure 5). Thus, we concluded that 2.4G2-mediated aggregation of FcγRIIB/FcγRIII affects the ability of FcγRIIB to co-localize with growing FcεRI clusters after Ag stimulation as it is less accessible to Ag-dependent redistribution. This was further supported by the observation that FcγRIIB ITIM phosphorylation progressively decreased with increasing 2.4G2 concentrations (Figure 5C). This emphasized the relevance of the spatial proximity between FcγRIIB and FcεRI even in a system in which FcγRIIB phosphorylation is independent of Ig-driven cross-linking.

We showed that FcγRIIB contributes to SHIP1 phosphorylation in an IgG-independent manner (Figure 3D) and that this process is based on the proximity of FcγRIIB and FcεRI (Figure 4A). FcεRI cross-linking induces the dose-dependent formation of a submembranous F-actin meshwork, which contributes to attenuation of MC activation at supra-optimal Ag concentrations (2–4). Inhibition of F-actin generation with latrunculin B (Lat. B) reduces tyrosine phosphorylation of SHIP1 and enhances degranulation as well as mobility of FcεRI clusters (3–5, 31). Thus, we investigated, whether the formation of F-actin also contributes to phosphorylation of FcγRIIB. Therefore, BMMCs were either incubated with 10 μM Lat. B or vehicle (DMSO) for 15 min prior to Ag stimulation (Figure 5D). The general pattern of FcγRIIB phosphorylation was not affected by Lat. B treatment, showing no measurable Y309 phosphorylation in unstimulated and optimally stimulated samples, but distinct bands after supra-optimal stimulation (Figure 5D). However, comparison of the supra-optimally challenged samples revealed that FcγRIIB ITIM tyrosine phosphorylation was significantly decreased by 42% (*p* = 0.0046) after Lat. B treatment compared to DMSO controls (Figure 5D). This finding is in line with previously reported reduction of SHIP1 phosphorylation after Lat. B treatment (5) and matches with a negative regulatory role of FcγRIIB.

### Loss of FcγRIIB affects Ca^2+^ mobilization and MC effector functions

Due to the close structural and functional interaction of FcγRIIB and SHIP1, we next analyzed the contribution of FcγRIIB to the control of MC effector responses upon FcεRI triggering in WT and *Fcgr2*^−/−^ BMMCs.

**Figure 6:**
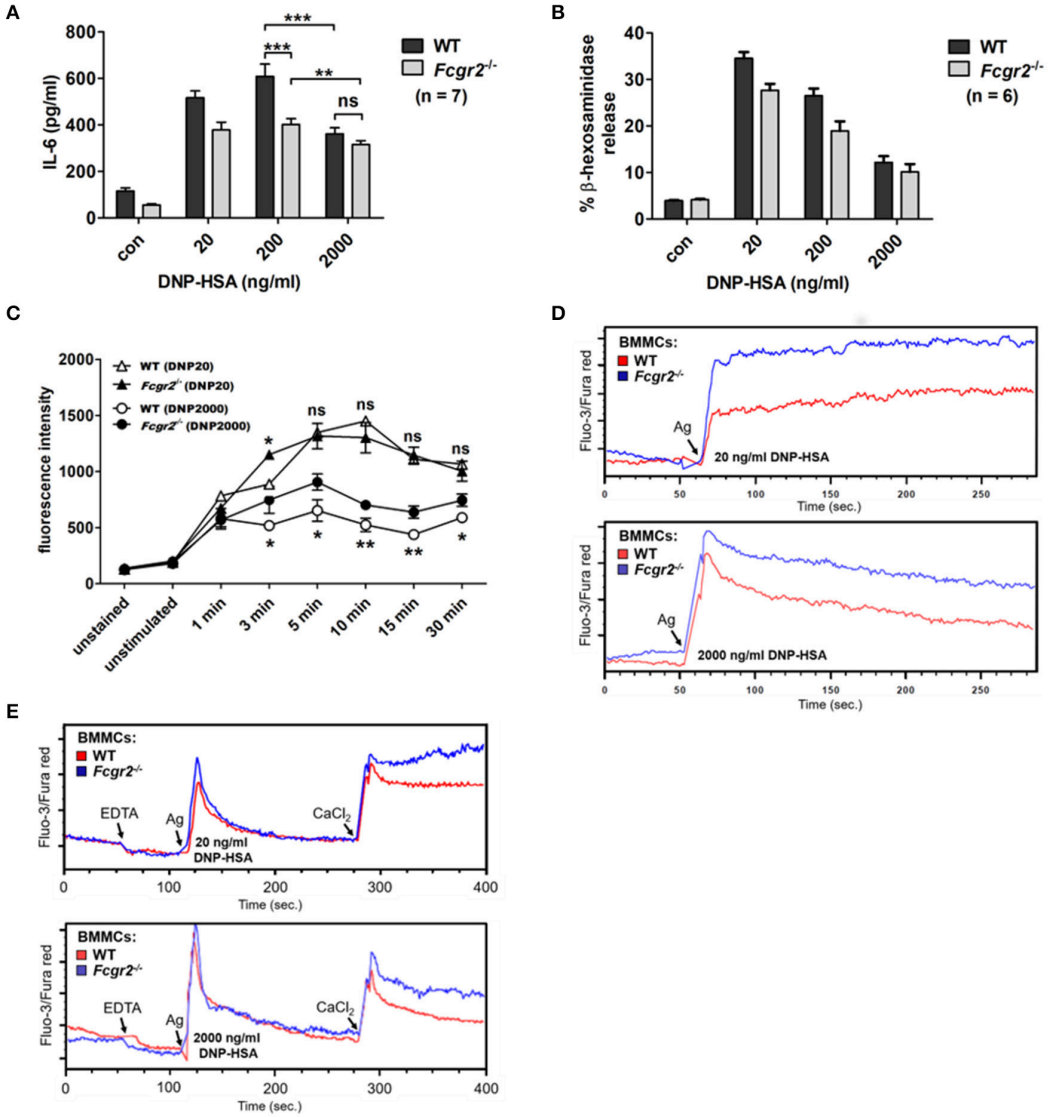
Altered activation phenotype in *Fcgr2*^−/−^ BMMCs. **(A)** Murine WT and *Fcgr2b*^−/−^ BMMCs were challenged with increasing concentrations of DNP-HSA for 4 h. IL-6 release in the culture supernatant was measured by ELISA. **(B)** WT and *Fcgr2*^−/−^ BMMCs were challenged with increasing Ag concentrations for 30 min and β-hexosaminidase release was measured. **(C)** LAMP-1 assay was performed to analyze differences in the kinetics of vesicle fusion between WT and *Fcgr2*^−/−^ BMMCs. Cells were stimulated as depicted, stained with a FITC-conjugated α-LAMP-1 antibody and analyzed by flow cytometry. **(D,E)** SPE-7 sensitized WT and *Fcgr2*^−/−^ BMMCs were stained with the Ca^2+^-sensitive fluorophores Fluo-3 and Fura Red. **(D)** Steady state fluorescence was measured for 1 min, to determine the Ca^2+^ signal in unstimulated cells. Indicated Ag concentrations were added and changes in cytosolic Ca^2+^ levels were measured for another 4 min. **(E)** Differentiation between intra- and extracellular Ca^2+^. Steady-state fluorescence in WT and *Fcgr2*^−/−^ MCs was measured for 1 min. 1 mM EDTA was added to chelate extracellular Ca^2+^ and the fluorescence signal was measured for another minute. After 2 min cells were stimulated with DNP-HSA at either 20 or 2000 ng/ml and the resulting increase in cytosolic Ca^2+^ was measured for 2.5 min. Finally, 2 mM CaCl_2_ was added to refill extracellular Ca^2+^ stores and the resulting SOC influx was measured for 2.5 min. Results shown in this figure are representatives of at least 3 independent experiments and in the diagrams depicted as mean ± SD. ^*^p < 0.05, ^**^p < 0.005, ^***^p < 0.0005.

Regarding production of the pro-inflammatory cytokine IL-6, WT MCs displayed the characteristic bell-shaped dose-response behavior, whereas the pattern for *Fcgr2*^−/−^ BMMCs appeared considerably altered with only minor differences in the responses between the increasing Ag concentrations used for stimulation (Figure 6A). After optimal stimulation (200 ng/ml of DNP-HSA), overall IL-6 release was significantly higher in WT compared to *Fcgr2*^−/−^ BMMCs. In contrast, Il-6 levels converged after supra-optimal stimulation (2,000 ng/ml DNP-HSA) of WT and *Fcgr2*^−/−^ BMMCs. This illustrates that the attenuation of IL-6 release in response to supra-optimal stimulation is less pronounced in *Fcgr2*^−/−^ compared to WT BMMCs.

Next, we compared Ag-triggered degranulation using two different techniques. First, the β-hexosaminidase assay revealed no enhanced mediator release in *Fcgr2*^−/−^ BMMCs (Figure 6B). Both WT and *Fcgr2*^−/−^ BMMCs showed a comparable dose-response behavior. Additionally, we determined the appearance of the lysosomal membrane protein LAMP-1 (CD107a) on the cell surface upon Ag-induced fusion of vesicular and plasma membrane by FACS. Interestingly, the comparison of degranulation kinetics by means of the LAMP-1 assay revealed slight but consistent differences. Under optimal conditions, slightly higher amounts of LAMP-1 appeared on the surfaces of WT compared to *Fcgr2*^−/−^ BMMCs. Contrary after supra-optimal stimulation, LAMP-1 levels were consistently increased in *Fcgr2*^−/−^ BMMCs, which indicates stronger vesicle fusion to the plasma membrane (Figure 6C; Supplementary Figures 6, 7).

Encouraged by this finding, we analyzed Ag-induced Ca^2+^ mobilization in these cells, since Ca^2+^ is a mandatory trigger for membrane fusion and thus degranulation (32). Indeed, Ca^2+^ mobilization was distinctly increased in *Fcgr2*^−/−^ BMMCs in response to both optimal as well as supra-optimal Ag concentrations (Figure 6D). In contrast to this, Ca^2+^/calcineurin/NFAT-dependent effector functions like IL-13 (Supplementary Figure 8) or TNF (Supplementary Figure 9) synthesis were not enhanced in *Fcgr2*^−/−^ BMMCs. Further degranulation, which also was expected to be influenced by increased Ca^2+^ mobilization, remained mostly unaltered (Figures 6B,C). Besides Ca^2+^, degranulation further depends on PI3K activation, which is not enhanced in comparison to WT BMMCs. Phosphorylation of AKT as a measure for PI3K activity remained unaltered in *Fcgr2*^−/−^ BMMCs (Supplementary Figure 10), which is consistent with largely unaffected degranulation. To differentiate between intracellular release of Ca^2+^ from the endoplasmic reticulum and influx of extracellular Ca^2+^ in a process called store-operated Ca^2+^ entry, we chelated extracellular Ca^2+^ by addition of EDTA prior to Ag stimulation (Figure 6E). After the release of intracellular Ca^2+^ and the subsequent return to base line (first transient peak) of the intracellular Ca^2+^ concentration, we restored extracellular Ca^2+^ levels by adding CaCl_2_ to measure Ca^2+^ influx through opened store-operated Ca^2+^ channels. We clearly observed that the differences in Ca^2+^ mobilization between WT and *Fcgr2*^−/−^ BMMCs were based on the influx of extracellular rather than the release of intracellular Ca^2+^ ions. In conclusion, our experiments showed partly enhanced MC activity in *Fcgr2*^−/−^ compared to WT cells, particularly upon supra-optimal stimulation, which indicated a negative regulatory function of FcγRIIB in response to FcεRI activation.

## Discussion

In this study, we have found that activation of the FcεRI by high, supra-optimal Ag concentrations causes co-aggregation{Formatting Citation} with the inhibitory FcγRIIB, resulting in LYN-dependent, SHIP1-stabilized ITIM phosphorylation of the FcγRIIB. Ag-induced tyrosine phosphorylation of the FcγRIIB was identified in a phospho-proteomic approach analyzing differences in protein tyrosine phosphorylation between optimally and supra-optimally stimulated BMMCs. This was motivated by our previous finding that the dominant negative regulator of MC activation, SHIP1, is phosphorylated strongest in response to high Ag concentrations (5).

FcγRIIB negatively regulates signaling of Fc- and B-cell receptors (33, 34), to which it is cross-linked via IgG-containing immune-complexes (14, 20). Co-aggregation leads to subsequent phosphorylation of the FcγRIIB ITIM by LYN, which promotes recruitment of SHIP1. Functional interaction between FcγRIIB and SHIP1 is well-documented (21, 29, 30).

In the present study, we observed passive co-localization of FcγRIIB and FcεRI that is independent of IgG-mediated receptor cross-linking. Instead it is Ag-mediated and progresses with increasing stimulus concentrations. Co-localization is associated with substantial phosphorylation of the FcγRIIB ITIM, which also increases in an Ag-dependent manner. In analogy to IgG-based activation (29), ITIM phosphorylation in this context is mediated by LYN and significantly contributes to recruitment of the negative regulator SHIP1. Upon stimulation, ITIM phosphorylation and consequently SHIP1 binding increases progressively. The lipid phosphatase is considered to be the main interaction partner of the FcγRIIB ITIM (35) and we show that it is not only recruited upon FcγRIIB phosphorylation, but it also affects ITIM phosphorylation itself. SHIP1 binding to the ITIM acts as a protective factor against dephosphorylation, as a loss of SHIP1 is accompanied by an essential decrease of ITIM phosphorylation, which can be counteracted with the inhibition of protein tyrosine phosphatases (PV treatment). This stresses the outstanding relevance of SHIP1 as binding partner for the FcγRIIB ITIM. Other potential interactors seem to have substantially less affinity to FcγRIIB, which results in immediate dephosphorylation of Y309 in the absence of SHIP1.

In our quantitative proteomics analysis especially Y326, but also Y309 within the ITIM of FcγRIIB displayed increased phosphorylation. Phosphorylated Y326 interacts with the SH2-domain of the adaptor protein GRB2, which, via its C-terminal SH3-domain, stabilizes the SHIP1-FcγRIIB interaction (36). This interaction itself is essentially mediated by the ITIM of FcγRIIB that – when tyrosine-phosphorylated—enables binding of SHIP1 via its single SH2-domain (35).

Besides FcγRIIB also other proteins displayed increased tyrosine phosphorylation after supra-optimal stimulation. In case of LAT2, Y59, and to a lesser extent Y98 were more strongly phosphorylated. Previous studies indicated a negative regulatory role for LAT2; thus, an increase in tyrosine phosphorylation at inhibitory Ag concentrations would be in line with these findings (37, 38). Some components of the cytoskeleton also showed enhanced tyrosine phosphorylation matching with the fact that cytoskeletal rearrangements mainly happen under supra-optimal conditions. Unexpectedly, our current findings with respect to tyrosine phosphorylation of SHIP1 did not reflect our previous results (5). In fact, the detected tyrosine phosphorylations in SHIP1 (Y868, Y1021, and Y1166) were only slightly enhanced (Y868 and Y1166) or even reduced (Y1021) after supra-optimal stimulation. However, in our previous work (5), immunoprecipitated SHIP1 was analyzed by anti-pTyr Western blotting and hence no information on single tyrosine residues can be deduced. Thus, the main LYN target tyrosine in SHIP1 phosphorylated under supra-optimal conditions is still to be discovered. Furthermore, increased phosphorylation was measured in the protein tyrosine phosphatase SHP-1. However, a study by Daëron and colleagues revealed that SHP-1 is not recruited by FcγRIIB *in vivo* (35). Also, the ITAM of the FcRγ chain was most strongly tyrosine phosphorylated after supra-optimal stimulation, which is surprising as FcRγ is closely linked to FcεRI activation. Nevertheless, increased phosphorylation at inhibitory Ag concentrations can be explained, as LYN, which also mediates phosphorylation of the FcRγ ITAM (39), is known to be most strongly active at high Ag concentrations (13). Further, passive recruitment of membrane receptors due to increasing FcεRI clusters might not be limited to FcγRIIB. FcγRIII that is also associated to FcRγ chains (40) could be recruited in a similar way, which would also explain increased phosphorylation of the FcRγ ITAM after supra-optimal stimulation.

In general, up- or down-regulation of tyrosine-phosphorylated proteins upon optimal vs. supra-optimal Ag stimulation was mostly rather weak (Supplementary Tables 1, 2). This is in line with previous findings by Gimborn et al., who observed increased tyrosine phosphorylations from low to optimal doses of Ag, but only subtle changes from optimal to supra-optimal Ag doses (5). Thus, the dose-response behavior of FcγRIIB Y309 phosphorylation certainly is not unique, however, nevertheless particular.

In contrast to Ig-based activation of FcγRIIB, no extracellular, directly “cross-linking agents” like immune complexes are involved in passive co-localization of FcγRIIB and FcεRI, which raises the question how receptors approach to each other in an IgG-independent setup. This spatial proximity is indispensable to sufficiently phosphorylate FcγRIIB, since LYN is associated to FcεRI (13, 41–43). In this context it is well-investigated that FcεRI-mediated stimulation is accompanied by extensive rearrangements of the actin cytoskeleton (4, 44, 45). A cage-like F-actin structure forms and condenses progressively with increasing Ag levels. The resulting restriction of membrane protein diffusion was suggested to have an important regulatory role in receptor signaling (31) and could also contribute to IgG-independent FcγRIIB recruitment. Different studies, by the inhibition of actin polymerization, showed the negative impact of the SMC on degranulation and FcεRI signaling (3–5). The use of Lat. B or similar reagents, which prevent actin polymerization, increased Ag-triggered degranulation in general, but most pronounced at supra-optimal conditions (5). In addition, phosphorylation of SHIP1 (5) and detergent insolubility of FcεRI were decreased (3). In line, we observed significantly decreased FcγRIIB phosphorylation after Lat. B treatment. Thus F-actin condensation most likely is involved in the recruitment, as it could facilitate co-localization of FcγRIIB and LYN/FcεRI.

Ultimately, one could envision the following model for passive, Ag-dependent recruitment of FcγRIIB: Upon FcεRI-mediated stimulation of MCs, polymerization of the F-actin cytoskeleton proceeds and FcεRI clusters grow with increasing Ag concentrations. Thus, also FcγRIIB is progressively confined and involved into these growing clusters. This causes proximity to LYN in an Ag-dependent manner and subsequent ITIM phosphorylation, resulting in SHIP1 recruitment. Eventually, FcγRIIB acts as an adaptor for SHIP1, contributing to the Ag-dependent membrane recruitment of the lipid phosphatase.

Loss of SHIP1 drastically increases different Ag-triggered MC effector functions like degranulation and cytokine production (5, 46). Thus, we assumed to see a comparably pronounced phenotype in FcγRIIB-deficient MCs, but changes in *Fcgr2*^−/−^ BMMCs were rather mild. In fact, supra-optimal cytokine production and extracellular Ca^2+^ influx was positively altered in *Fcgr2*^−/−^ BMMCs, whereas degranulation was hardly affected (Figure 6). Slightly enhanced cytokine synthesis could be explained by reduced SHIP1 recruitment, as SHIP1 via its lipid phosphatase activity down-regulates PIP_3_ generated by PI3K upon FcεRI stimulation (8). This in turn could cause increased activation of various PH-domain-containing proteins being recruited to the plasma membrane in a PIP_3_-dependent manner, such as AKT. Interestingly, FcεRI-mediated activation of PLCγ has been shown to be independent of PI3K (47), excluding enhanced PLCγ activation as the reason for the observed Ca^2+^ phenotype in *Fcgr2*^−/−^ BMMCs. Additionally, sphingosine kinase was demonstrated to significantly contribute to Ag-triggered Ca^2+^ flux (48), however, its control by PI3K/SHIP1 is not known so far.

Unexpectedly, Ca^2+^/calcineurin/NFAT-dependent mediators like IL-13 (Supplementary Figure 8) or TNF (Supplementary Figure 9) were not increased in *Fcgr2*^−/−^ BMMCs. Also phosphorylation of AKT remained unaltered in *Fcgr2*^−/−^ BMMCs (Supplementary Figure 10), whereas it is strongly increased in *Ship1*^−/−^ BMMCs (5). This finding goes in line with the mostly unaffected degranulation that depends on both Ca^2+^ mobilization and PI3K activation. It appears that the residual level of SHIP1 phosphorylation, which most likely can be taken as a measure for SHIP1 activity (9), can sufficiently maintain the bell-shaped dose-response for degranulation that is lost in *Ship1*^−/−^ BMMCs (5). Although the membrane protein FcγRIIB by binding to SHIP1 significantly contributes to SHIP1 membrane recruitment, it undoubtedly is only one among various, redundantly acting proteins, which clearly stresses the importance of SHIP1 (and its membrane recruitment) as a central negative regulator of FcεRI signaling.

One other candidate in this context is FcεRIβ, which was frequently reported to be most strongly tyrosine-phosphorylated upon supra-optimal stimulation (5, 13, 49) and to interact with SHIP1 via its unique ITAM sequence (13, 50). We were able to co-precipitate FcεRIβ with SHIP1 from total cell lysates of WT and *Fcgr2*^−/−^ BMMCs (Supplementary Figure 11), whereby in FcγRIIB-deficient MCs co-precipitation was more pronounced after supra-optimal stimulation compared to WT cells. Enhanced interaction between FcεRIβ and SHIP1 might be a consequence of the FcγRIIB deficit, as its highly affine ITIM is not available for the interaction with SHIP1. This might enable enhanced SHIP1 recruitment via FcεRIβ. The increase in the number of SHIP1-FcεRIβ complexes in the absence of FcγRIIB is a clear indicator for the tight back-up control of SHIP1 recruitment. As a central negative regulator of MC function, SHIP1 strongly contributes to homeostasis. As such its loss or loss of function can potentially harm the cell. Thus, proteins acting redundantly to FcγRIIB may ensure its functionality. Moreover, we co-precipitated leukocyte immunoglobulin-like receptor B3 (LIR-3) that also contains ITIMs with SHIP1 from whole cell lysates of both, WT and *Fcgr2*^−/−^ BMMCs (Supplementary Figure 11). LIR-3 was previously described to attenuate FcεRI-mediated activation of basophils (51), which makes it a further potential membrane anchor for SHIP1 recruitment.

In conclusion, following an unbiased MS approach focusing on proteins that are most strongly tyrosine-phosphorylated upon supra-optimal Ag stimulation, we found IgG-independent co-aggregation of FcεRI and FcγRIIB. In this context, our data established the LYN/FcγRIIB/SHIP1 signalosome in response to FcεRI stimulation. The fact that SHIP1 tyrosine phosphorylation/activation was significantly attenuated, however not abrogated in FcγRIIB-deficient BMMCs, speaks for the necessity of tightly controlled backup regulation of SHIP1, the gatekeeper of MC activation.

## Materials and methods

### Animals and cell culture

Bone marrow-derived mast cells (BMMCs) were generated as described previously (52). The quoted procedure was applied in this study to generate *Ship1*^−/−^ (129/Sv × C57Bl/6), *Lyn*^−/−^(129/Sv × C57Bl/6), *Lyn*^−/−^ *Ship1*^−/−^ (129/Sv × C57Bl/6), and *Fcgr2*^−/−^ (C57Bl/6) BMMCs. To each the related WT BMMCs were generated according to the same protocol. Experiments were performed in accordance with German legislation governing animal studies and following the principles of laboratory animal care. Mice are held in the Institute of Laboratory Animal Science, Medical Faculty of RWTH Aachen University. The institute holds a license for husbandry and breeding of laboratory animals from the veterinary office of the Städteregion Aachen (administrative district). The institute follows a quality management system, which is certified according to DIN ISO 9001/2008. Every step in this project involving mice was reviewed by the animal welfare officer. All experiments were approved by the Landesamt für Natur, Umwelt, und Verbraucherschutz NRW (LANUV), Recklinghausen (AZ 84-02.04.2016.A496).

### β-hexosaminidase assay

To measure degranulation, BMMCs preloaded with 0.15 μg/ml IgE (SPE-7; Sigma) overnight (37°C, 5% CO_2_) were washed in sterile PBS, resuspended in Tyrode's buffer (130 mM NaCl, 5 mM KCl, 1.4 mM CaCl_2_, 1 mM MgCl_2_, 5.6 mM glucose, and 0.1% BSA in 10 mM HEPES, pH 7.4) and adapted to 37°C. Cells were stimulated with different concentrations of Ag (DNP-HSA; Sigma) for 30 min at 37°C. The degree of degranulation was determined by measuring β-hexosaminidase release (53).

### Cytokine elisa

BMMCs were preloaded with 0.15 μg/ml IgE (SPE-7) overnight (37°C; 5% CO_2_)_._ Cells were washed in sterile PBS and adjusted to 1.2 × 10^6^/ml in RPMI 1640 containing 0.1% BSA. Cells were stimulated with different concentrations of Ag (DNP-HSA; Sigma) for 4 h at 37°C Mouse IL-6 ELISA (BD Pharmingen) was performed according to the manufacturer's instructions. Levels of cytokines varied between experiments due to age of the cells. Qualitative differences or similarities between WT and mutant cells, however, were consistent throughout the study.

### Flow cytometry

BMMCs were stained with FITC-conjugated anti-FcεRIα IgG (eBioscience) and PE-conjugated anti-CD16/CD32 IgG (BD) for 25 min at 4°C in FACS buffer (1 × PBS, 3% FCS, 0.1 % sodium azide). Cells were analyzed by flow cytometry using a FACSCanto II (BD).

For experiments using Fc-block, MCs were differentially loaded with IgE and incubated with 2.4G2 (BD) as described in the respective experiments. Afterwards cells were stained with anti-IgE IgG (SouthernBiotech) and analyzed by flow cytometry.

To perform the LAMP-1 assay, BMMCs preloaded with 0.15 μg/ml SPE-7 overnight (37°C; 5% CO_2_) were washed in sterile PBS, resuspended in RPMI 1640 containing 0.1% BSA and adapted to 37°C. MCs were stimulated with the indicated concentrations of DNP-HSA for the indicated periods. MCs were pelleted, washed with FACS buffer and stained with FITC-conjugated anti-LAMP1 immunoglobulins (BioLegend) for 25 min at 4°C in FACS buffer. Cells were then washed twice, resuspended in FACS-buffer, and LAMP-1 surface expression was determined by flow cytometry. Data were analyzed using the FlowJo analysis software (Tree Star).

### Calcium measurement

BMMCs were preloaded with 0.15 μg/ml IgE (SPE-7) overnight, washed with sterile PBS and resuspended at 1 × 10^7^ cells/ml in RPMI 1640 containing 0.1% BSA. Calcium indicators Fluo-3 (1.3 μM) and Fura Red (2.75 μM), as well as 5 μl/ml of 20% pluronic F-127 in DMSO (all Thermo Fisher) were added, and cells were incubated for 45 min at 37°C. MCs were pelleted, resuspended in RPMI 1640 containing 0.1% BSA, and analyzed in a FACSCalibur (BD Biosciences) with the indicated stimulation procedures. Intracellular calcium release was recorded in the presence of 1 mM EDTA (Sigma). For data analysis the FlowJo analysis software (Tree Star) was used. FACS profiles were converted to line graphs.

### Proximity ligation assay (PLA)

Cover slips were incubated with 12.5 μg/ml poly-L-lysine for 1 h, washed twice with sterile PBS and dried. An area of approximately 1 cm^2^ was edged with a hydrophobic pen (Sigma) to limit the reaction area for PLA reagents. IgE-preloaded BMMCs (0.5 μg/ml of SPE-7, overnight) were washed in sterile PBS and resuspended in RPMI/0.1% BSA at 3.5 × 10^6^ cells/ml. 1 ml of the cell suspension was added to each cover slip. Cells were incubated at 37°C for 1 h and centrifuged on the cover slips at 1,200 rpm for 5 min at 4°C. Supernatants were aspirated. Temperature was carefully adjusted to 37°C for 20 min with 1 ml of pre-warmed medium. MCs were stimulated with the indicated concentrations of DNP-HSA for 2 min. Cover slips were washed twice with PBS^++^ (1 × PBS, 1 mM MgCl_2_, 0.1 mM CaCl_2_). Cells were fixed with 4% paraformaldehyde (PFA) for 10 min in the dark at room temperature (RT) followed by another washing step with PBS^++^. After quenching with 50 mM NH_4_Cl for 5 min (RT), cells were washed with PBS^++^. Samples were further treated according to the instructions provided with the Duolink^®;^ *in situ* Starter Kit (Sigma) and finally analyzed using a Zeiss LSM 710 (confocal laser scanning microscope). Signal calculations were done with BlobFinder (The Centre for Image Analysis at Uppsala University, Sweden).

### Confocal laser scanning microscopy

BMMCs were pretreated as indicated in the respective experiment and concentrated in PBS^++^ at 3.5 × 10^6^ cells/ml. For each sample 1 ml of the cell suspension was added to a cover slip and centrifuged at 1,200 rpm for 7 min (4°C). Supernatant was aspirated, and cells were fixed with 4% PFA for 10 min in the dark (RT) followed by a washing step with PBS^++^. After quenching with 50 mM NH_4_Cl for 5 min at RT, cells were washed with PBS^++^ and blocked with 5% BSA in PBS^++^ for 1 h at RT. Antibodies were diluted in 0.2% BSA-PBS^++^ according to the manufacturer's instructions and incubation with primary/secondary antibodies was conducted for 1.5 h at RT in the dark. If primary and secondary antibodies were used for staining, three washing steps were performed in between with 0.2% BSA-PBS^++^. After staining cells were washed three times in 0.2% BSA-PBS^++^. Nuclei were stained with DAPI (Sigma) (1:1000) for 10 min and cells were washed three times as described before. Cover slips were dried and mounted on microscope slides. Samples were analyzed using a Zeiss LSM 710.

### Mast cell stimulation, western blotting, and immunoprecipitation

IgE-preloaded BMMCs (0.15 μg/ml SPE-7, overnight) were washed in sterile PBS and resuspended in RPMI/0.1% BSA. Cells were stimulated with the indicated concentrations of Ag or monoclonal rat anti-mouse IgE antibody (Southern Biotech, clone 23G3, isotype IgG_1_*K*), respectively for indicated periods, pelleted and solubilized in phosphorylation solubilization buffer (PSB) (54) containing 0.5% NP-40 and 0.1% sodium deoxycholate at 4 °C. Lysates were subjected to SDS-PAGE and Western blot analysis as described previously (5). For immunoprecipitations the respective antibody was added to the lysates according to the manufacturer's recommendations after adjustment of protein amounts. Protein G sepharose beads (Sigma) were added and lysates were incubated on a rotator at 4°C overnight. Supernatants were removed. Precipitates were washed three times with PSB and analyzed by SDS-PAGE and Western blotting. For mass spectrometry analysis of IPs, precipitates were separated by SDS-PAGE. Gels were stained with Coomassie Brilliant Blue. Single bands were cut out and subjected to mass spectrometry analysis (55). Antibodies against SHIP-1 (N1 and P1C1), and CD32B were obtained from Santa Cruz Biotechnology, against P-Tyr (P-Tyr-100), P-PKB, and p85 from Cell Signaling Technology and anti-P-CD32 was purchased from LifeSpan BioSciences.

For the preparation of pervanadate solution, sodium orthovanadate (Na_3_VO_4_) was dissolved in distilled water (100 mM). Next pH was adjusted to 10. The solution was boiled up until the yellow color had fully disappeared and pH was readjusted to 10. If the color turned yellow again, the process of boiling and subsequent readjustment of pH was repeated until the solution remained colorless at pH 10. Pervanadate (PV) was prepared immediately before use by mixing 100 mM sodium orthovanadate and hydrogen peroxide in a molar ratio of 2:1 (H_2_O_2_:orthovanadate).

### Quantitative proteomics of P-Tyr containing peptides

100 million BMMCs per sample were loaded with 0.15 μg/ml IgE overnight. After stimulation with either an optimal (20 ng/ml) or a supra-optimal (2,000 ng/ml) concentration of DNP-HSA (one batch was left unstimulated as a control), cells were pelleted and resuspended in lysis buffer (8 M urea, 50 mM ammonium bicarbonate, 1 mM sodium pervanadate (pH 10), 20 μg/ml Aprotinin, 4 μg/ml Leupeptin and 1 mM PMSF). After sonification at 4°C (3 × 1 min per sample), cells were lysed for 30 min on ice. Lysates were cleared by centrifugation (30 min, 16,000 × G, 4°C) and overall protein amounts were determined and adjusted. Samples were reduced wit 2 mM dithiothreitol (DTT) (Roth) at 56°C for 30 min and alkylated with 4 mM iodoacetamide (IAA) (Sigma) for 25 min at RT in the dark. Next, proteins were digested with the lysyl endopeptidase Lys-C (Wako) (3 μg/mg of total protein) for 4 h (37°C). Samples were then diluted from 8 to 2 M urea with 50 mM ammonium bicarbonate and digested with trypsin (Thermo Fisher) (16 μg/mg of total protein) overnight. For stable isotope dimethyl labeling the on-column method described by Boersema et al. was used (16). After vacuum centrifugation, dried samples were resuspended in 333 μl of cold IP buffer (50 mM Tris, 150 mM NaCl, and 1% n-Octyl-β-D-glucopyranoside (NOG), pH 8.8), combined to one single sample and subjected to anti-P-Tyr immunoprecipitation. Then pH was adjusted to 7.4 by addition of 0.5 M Tris (pH 8.8). Anti-P-Tyr IP was conducted overnight with protein G sepharose beads that were covalently cross-linked to 4G10 anti-P-Tyr antibodies (Sigma) in advance (55). Divergent from the reference, protein G instead of protein A sepharose was used. Additionally, the amount of antibody was increased to 150 μg per 50 μl of packed beads, to enhance the efficiency of the P-Tyr IP. Precipitates were washed twice with IP buffer pH 7.4 and once with water. Peptides were eluted twice using 0.15% trifluoroacetic acid (TFA). The eluted peptides were concentrated as well as desalted using home-made C18 tips and dried by vacuum centrifugation. Finally, the sample was resuspended in 10% formic acid (FA) and subjected to mass spectrometry analysis.

The peptides were trapped on a C18 precolumn (Acclaim PepMap100, C18, 5 μm, 100 Å, 300 μm i.d. × 5 mm, Thermo Scientific) using buffer A (0.1% FA). This was followed by peptide separation on an analytical column (Acclaim PepMap100, C18, 5 μm, 100 Å, 75 μm i.d. × 25 cm, Thermo Scientific) by employing a 260 min or 280 min gradient from 5-40% buffer B (80% acetonitrile, 0.1% FA) at 230 nl/min. The eluent was sprayed into the mass spectrometer via stainless steel emitters using a spray voltage of 1.9 kV. Mass spectrometry was performed using an Orbitrap Elite (Thermo Scientific). The instrument was operated in data-dependent mode. Full-scan spectra were recorded in the Orbitrap (m/z 350 to m/z 1,500; resolution: 120,000; AGC: 5e5 ions). The top 10 precursors of each full scan were selected for fragmentation using CID in the ion trap (collision energy of 35%; 20 s dynamic exclusion range). All immunoprecipitations were analyzed in duplicate (technical replicate). The third experiment was carried out using a “label swap.” The resulting raw data files were analyzed by MaxQuant and the built-in Andromeda search engine (56, 57). The mouse Uniprot database version 06/18 (only canonical and reviewed entries) was used for protein identification. Search settings were: multiplicity of 3; labels: DimethLys0 and DimethNter0 for “light,” DimethLys4 and DimethNter4 for “intermediate,” and DimethLys8 and DimethNter8 for “heavy”; carbamidomethylation (Cys) as a fixed modification; oxidation (Met); phosphorylation (Ser, Thr, Tyr) as variable modifications. FDR 0.01% on protein, peptide and PTM level. Other MaxQuant parameters were left at their respective default settings. The result file was analyzed with Perseus (https://www.ncbi.nlm.nih.gov/pubmed/27348712) (version 1.5.5.3): peptides corresponding to reverse and contaminant protein entries were removed; a minimum phosphorylation site localization score of 0.75 was set as the minimum; only phosphorylated tyrosine residues were considered. To be included in the result file, a phosphorylation site had to be identified at least once in both technical replicates of one experiment with all ratios. The mean of all normalized ratios (each experiment and each technical replicate) was calculated and is reported in the Supplementary Tables 1,2. All ratios (20/control, 2,000/control, and 2,000/20) had to be detected in both an experiment and its corresponding technical replicate in order to be used for the calculation of the mean.

### Statistical analysis

All values are expressed as mean ± SD of n observations (n indicated in the respective figure legends). *p*-values were calculated by the unpaired two-tailed Student's *t*-test. For normalized data the one sample *t*-test including FDR (false discovery rate) was used. *p*-values of ^*^p < 0.05, ^**^p < 0.005, and ^***^p < 0.0005 were considered statistically significant. Values higher than a *p*-value of 0.05 were regarded as not significant.

## Data availability statement

The datasets generated and analyzed for this study are either included in the manuscript and the supplementary files or will be made available by the authors on request to any qualified researcher. The mass spectrometry proteomics data have been deposited to the ProteomeXchange Consortium (http://proteomecentral.proteomexchange.org) via the PRIDE partner repository (58) with the dataset identifier PXD009318.

## Author contributions

MG carried out experimental design, performed experiments, analyzed data and wrote the manuscript. CP carried out experimental design, performed mass spectrometry experiments, analyzed the respective data and provided experimental and analytical guidance. FN provided fundamental material. MH conceived the study, carried out experimental design, analyzed data and wrote the manuscript. All authors reviewed the manuscript.

### Conflict of interest statement

The authors declare that the research was conducted in the absence of any commercial or financial relationships that could be construed as a potential conflict of interest.
