# Supplementary material for: IgG-Independent Co-aggregation of FcεRI and FcγRIIB Results in LYN- and SHIP1-Dependent Tyrosine Phosphorylation of FcγRIIB in Murine Bone Marrow-Derived Mast Cells
Source: Front Immunol. 2018 Aug 27;9:1937. doi: 10.3389/fimmu.2018.01937 (PMC6119721; doi:10.3389/fimmu.2018.01937)
Supplement: Supplementary file 1 [file Data_Sheet_1.docx]

Supplementary Material

IgG-independent co-aggregation of FcεRI and FcγRIIB results in LYN- and SHIP1-dependent tyrosine phosphorylation of FcγRIIB

Mathias Gast^1^, Christian Preisinger^2^, Falk Nimmerjahn^3^, and Michael Huber^1*^

^1^Institute of Biochemistry and Molecular Immunology, Medical Faculty, RWTH Aachen University, Aachen, Germany

^2^ Proteomics Facility, IZKF, RWTH Aachen University, Aachen, Germany

^3^Institute of Genetics at the Department of Biology, Friedrich-Alexander-University Erlangen-Nürnberg, Erlangen, Germany

***Correspondence:** [mhuber@ukaachen.de](mailto:mhuber@ukaachen.de)

# Supplementary Figures


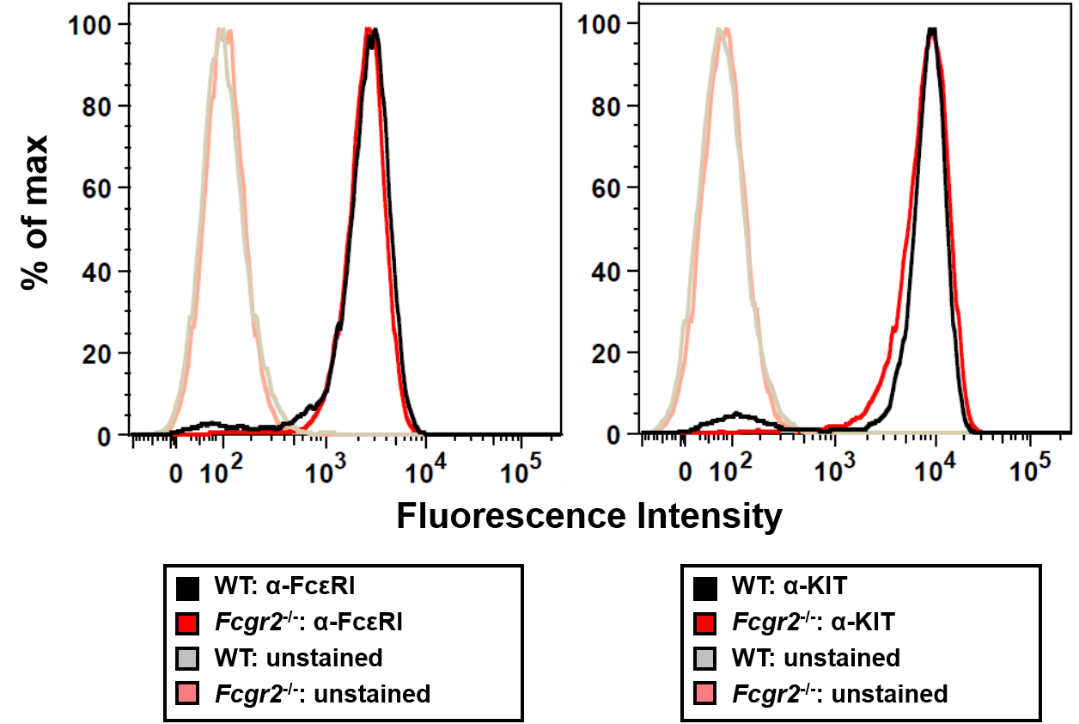


**Supplementary Figure S1:** WT and *Fcgr2*^-/-^ BMMCs were stained with FITC-labeled anti-FcεRI antibody (left panel) and PE-labeled anti-KIT antibody (right panel). Faint red and grey histograms show autofluorescence. Flow cytometry analysis to determine expression of the respective proteins on the cell surface showed that levels were comparable in WT and *Fcgr2*^-/-^ BMMCs.


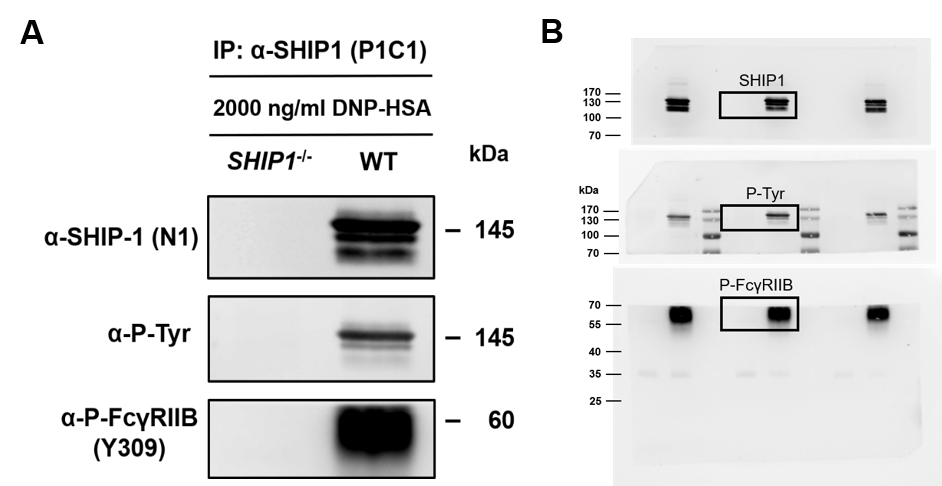


**Supplementary Figure S2:** Negative control for SHIP1 immunoprecipitation. **(A)** WT and *Ship1^-/-^* BMMCs were sensitized with SPE-7 overnight (0.15 µg/ml). Cells were challenged with 2000 ng/ml of DNP-HSA for 2 min. Immunoprecipitation against SHIP1 was conducted. Specificty of the IP with the respective anti-SHIP1 antibody (P1C1) was proven by the negative control. **(B)** Original blots belonging to bands shown in (A).


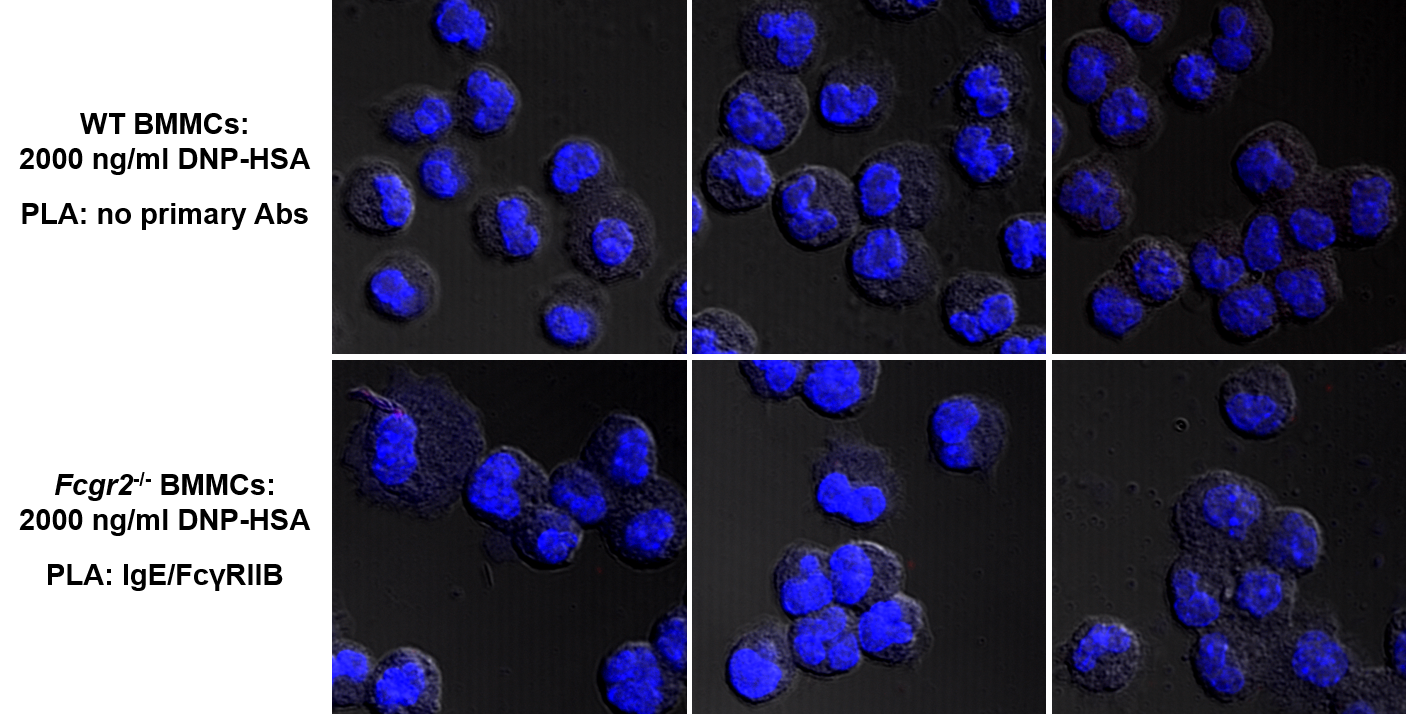


**Supplementary Figure S3:** Alternative negative control for *in situ* PLA shown in Fig.4. WT BMMCs were incubated with the solvent of the respective primary antibodies only and *Fcgr2^-/-^* BMMCs with both primary antibodies.


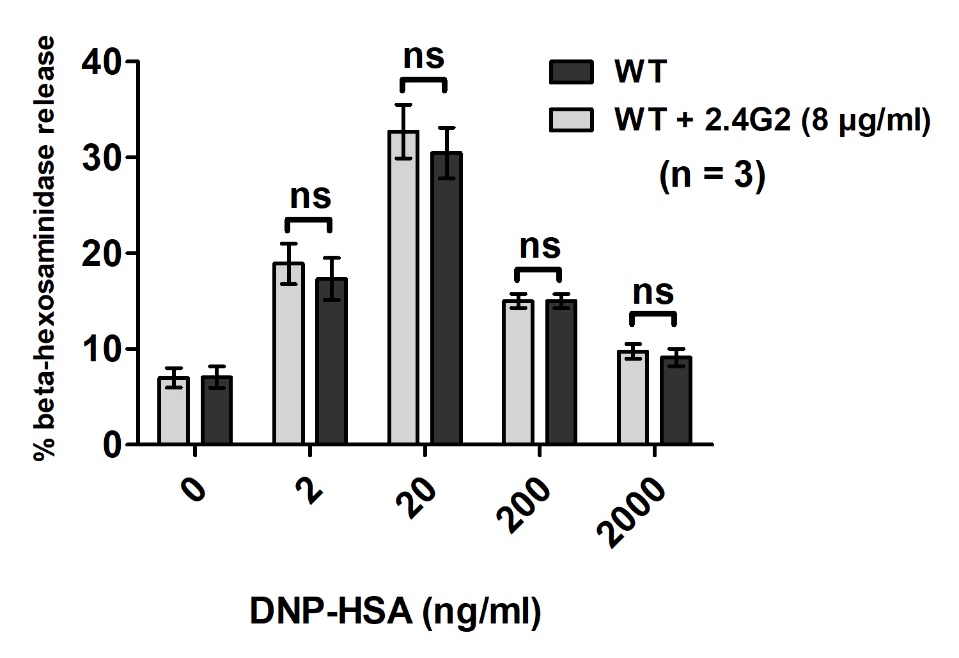


**Supplementary Figure S4:** WT BMMCs were sensitized with 0.15 µg/ml of SPE-7 overnight. Cells were split into two batches and treated with 8 µg/ml of 2.4G2 for 40 min or left untreated intstead. Cells were then stimulated with the respective amounts of DNP-HSA for 30 min and β-hexosaminidase release was determined.


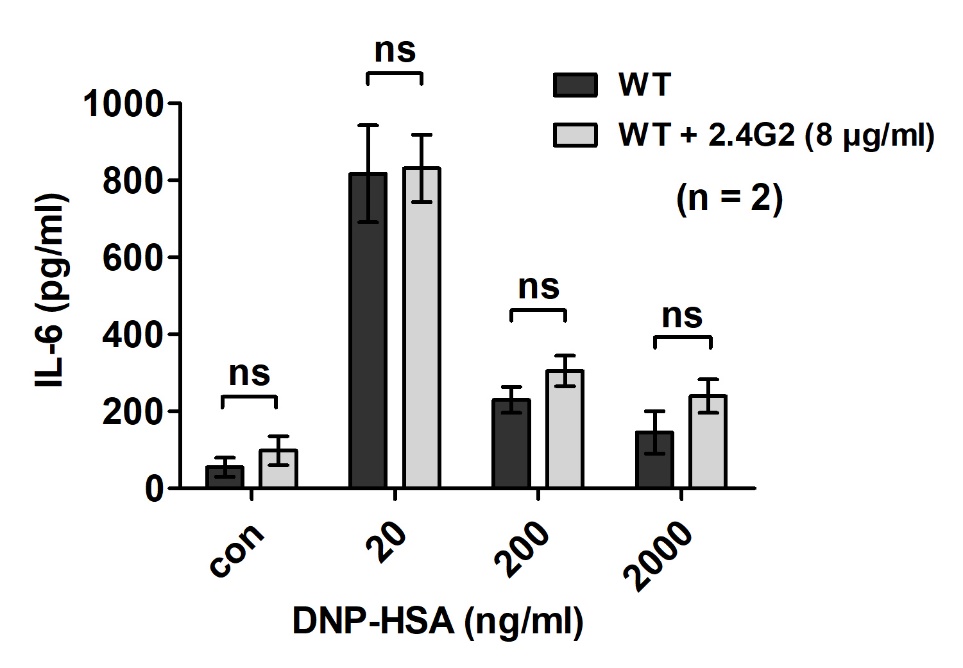


**Supplementary Figure S5:** WT BMMCs were sensitized with 0.15 µg/ml of SPE-7 overnight. Cells were split into two batches and treated with 8 µg/ml of 2.4G2 for 40 min or left untreated intstead. Cells were then stimulated with the respective amounts of DNP-HSA for 4 h and IL-6 release was determined.


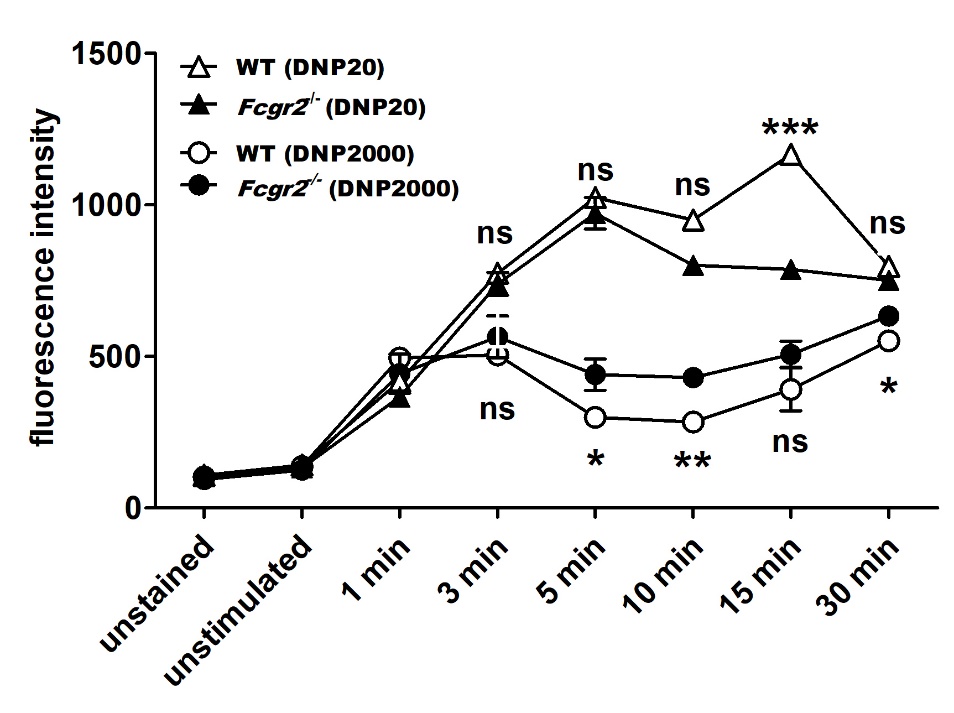


**Supplementary Figure S6:** LAMP-1 assay in WT and *Fcgr2*^-/-^ BMMCs. MCs were stimulated as depicted, stained with a FITC-conjugated α-LAMP-1 antibody and analyzed by flow cytometry. Vesicle fusion appeared to be increased upon supra-optimal stimulation in *Fcgr2*^-/-^, whereas after optimal stimulation it was partly enhanced in WT BMMCs.


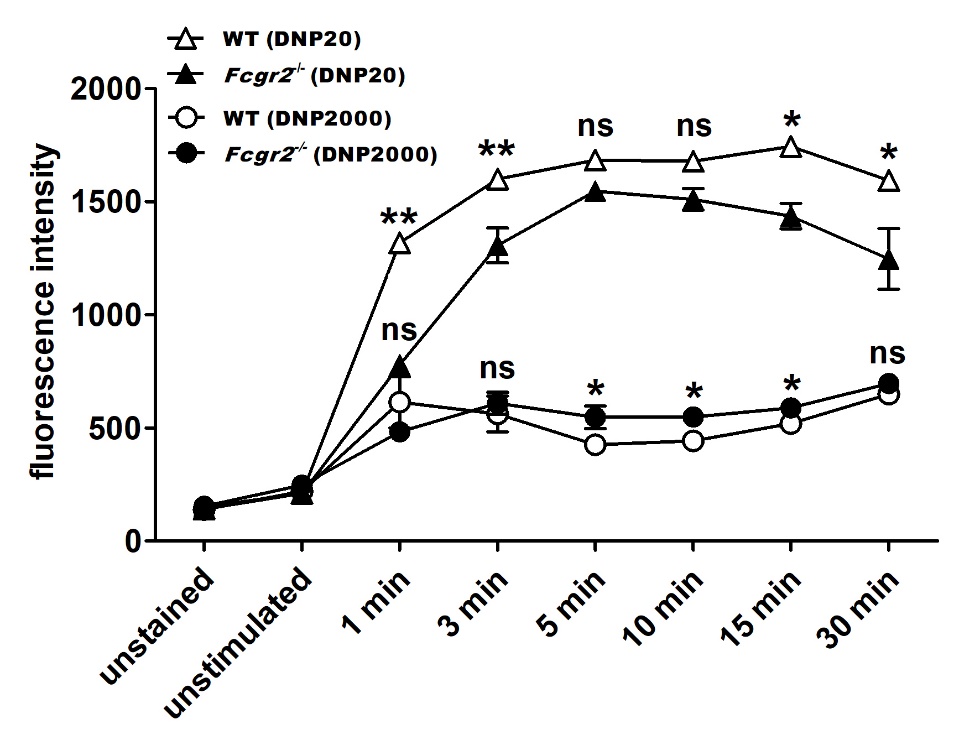


**Supplementary Figure S7:** LAMP-1 assay in WT and *Fcgr2*^-/-^ BMMCs. MCs were stimulated as depicted, stained with a FITC-conjugated α-LAMP-1 antibody and analyzed by flow cytometry. Vesicle fusion appeared to be increased upon supra-optimal stimulation in *Fcgr2*^-/-^, whereas after optimal stimulation it was enhanced in WT BMMCs.


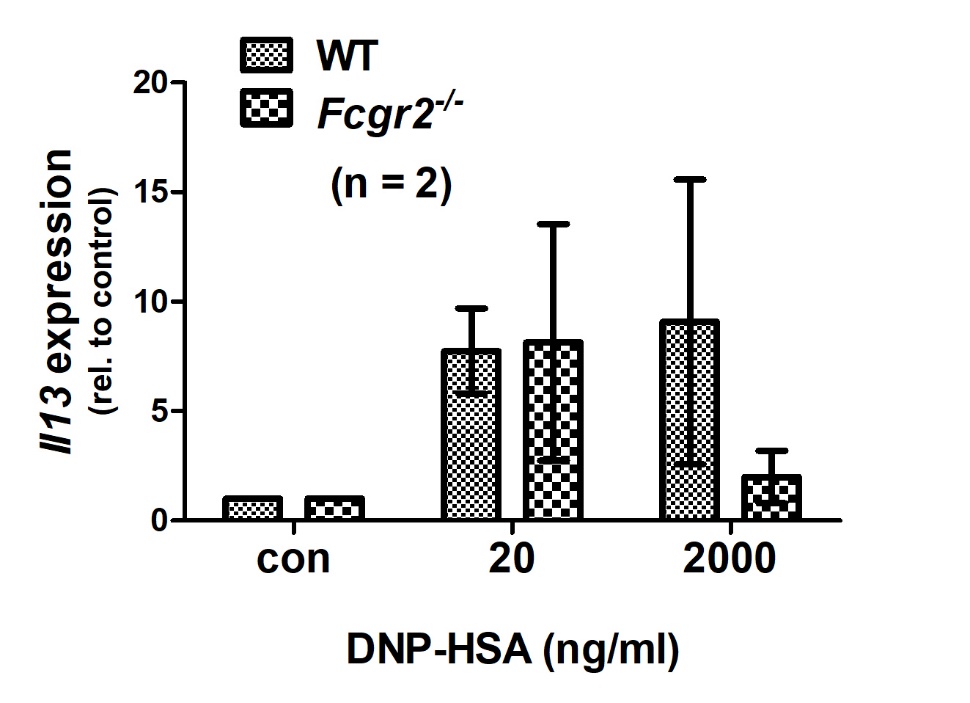


**Supplementary Figure S8:** *Il13* gene expression was determined in WT and *Fcgr2*^-/-^ BMMCs. MCs were stimulated as depicted for 2 h. After mRNA preparation, qPCR was conducted as described previously (1). WT and *Fcgr2*^-/-^ BMMCs did not display marked differences in *Il13* gene expression.


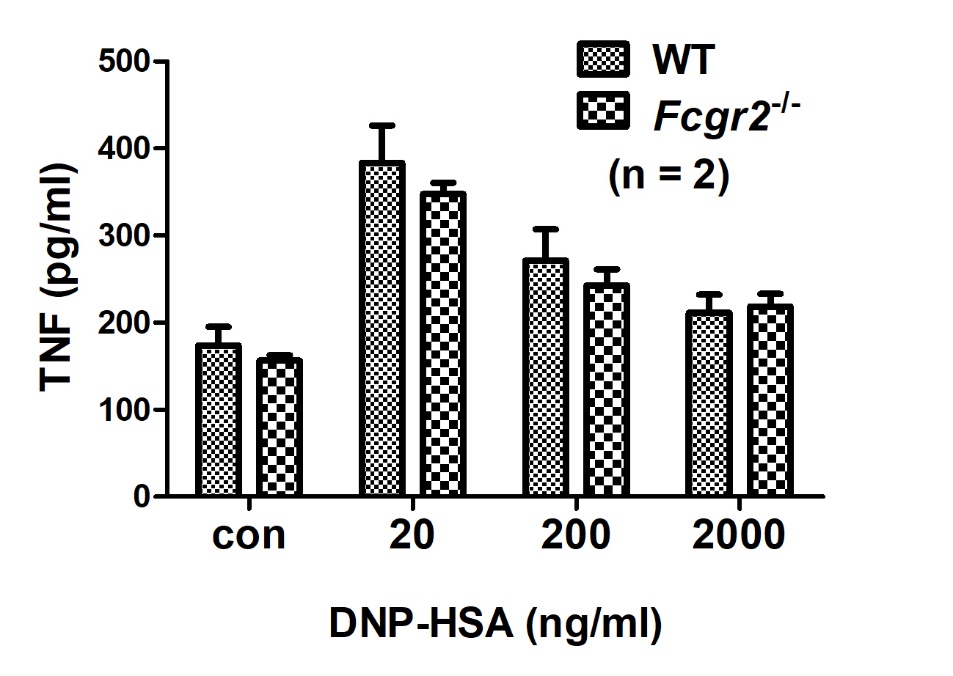


**Supplementary Figure S9:** TNF expression was determined in WT and *Fcgr2*^-/-^ BMMCs. MCs were stimulated as depicted for 3 h and TNF ELISAs (R&D Systems) were conducted according to the manufacturer’s instructions. TNF levels in *Fcgr2*^-/-^ BMMCs were equal to those measured in the respective WT.


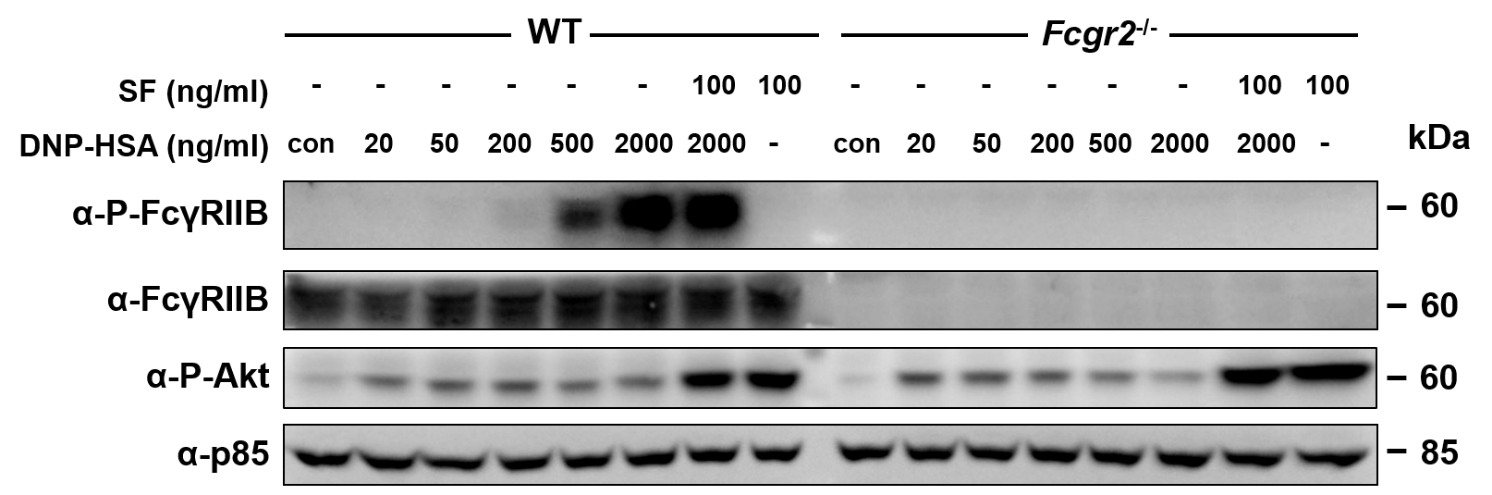


**Supplementary Figure S10:** SPE-7 sensitized (0.15 µg/ml) WT and *Fcgr2*^-/-^ BMMCs were stimulated as indicated in the figure. Stem cell factor (SF) stimulation was performed for 1 min, followed by subsequent stimulation with DNP-HSA for 2 min. Total cell lysates of MCs were then subjected to SDS-PAGE and Western blotting. WT BMMCs displayed increasing phosphorylation of FcγRIIB between 500 and 2000 ng/ml DNP-HSA. Additional SF stimulation before supra-optimal Ag challenge (2000 ng/ml DNP-HSA) did not affect FcγRIIB phosphorylation. Single challenge with 100 ng/ml of SF did not trigger FcγRIIB phosphorylation. Levels of Akt phosphorylation were similar in WT and *Fcgr2^-^*^/-^ BMMCs.


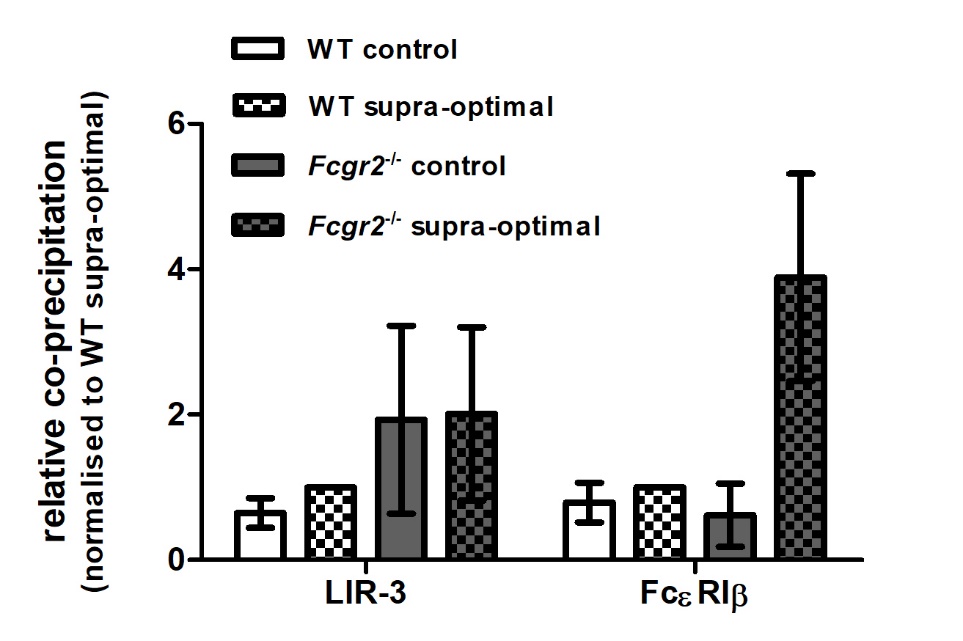


**Supplementary Figure S11:** Co-precipitation of LIR-3 and FcεRIβ with SHIP1 from whole cell lysates. WT and *Fcgr2*^-/-^ BMMCs were sensitized with SPE-7 (0.15 µg/ml) overnight. Cells were stimulated with a supra-optimal concentration of DNP-HSA (2000 ng/ml) or left unstimulated as a control. Cells were lysed and subjected to α-SHIP1 immunoprecipitation. Protein amounts within precipitates were compared by LC-MS and relative levels of LIR-3 and FcεRIβ co-precipitation were calculated. Values for LIR-3 and FcεRIβ were normalized to those obtained from WT BMMCs after stimulation with 2000 ng/ml DNP-HSA. While LIR-3 levels are equal in WT and *Fcgr2*^-/-^ BMMCs, FcεRIβ co-precipitation with SHIP1 seems to be increased in *Fcgr2*^-/-^ BMMCs after supra-optimal stimulation. Values are shown as mean ± SD (n = 2).


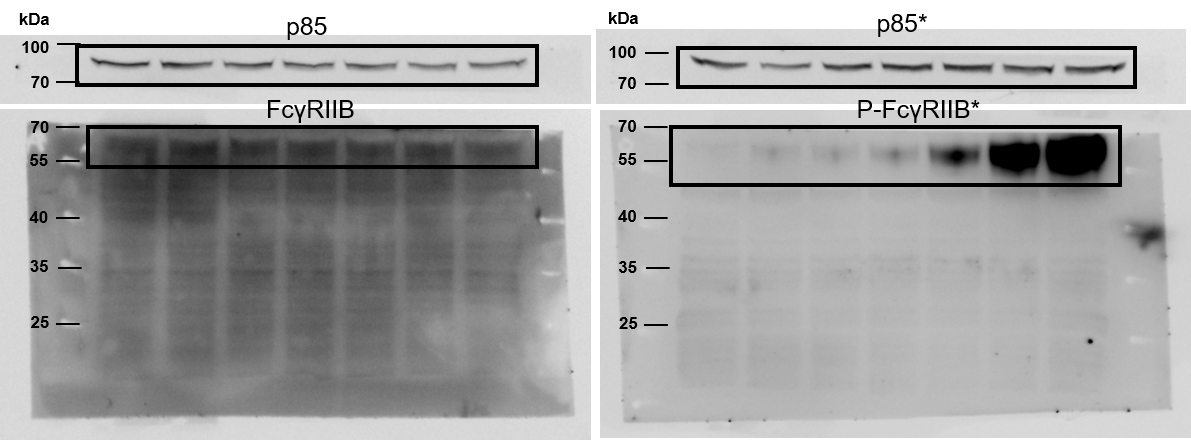


**Supplementary Figure S12:** Original blots belonging to bands shown in figure 2A.


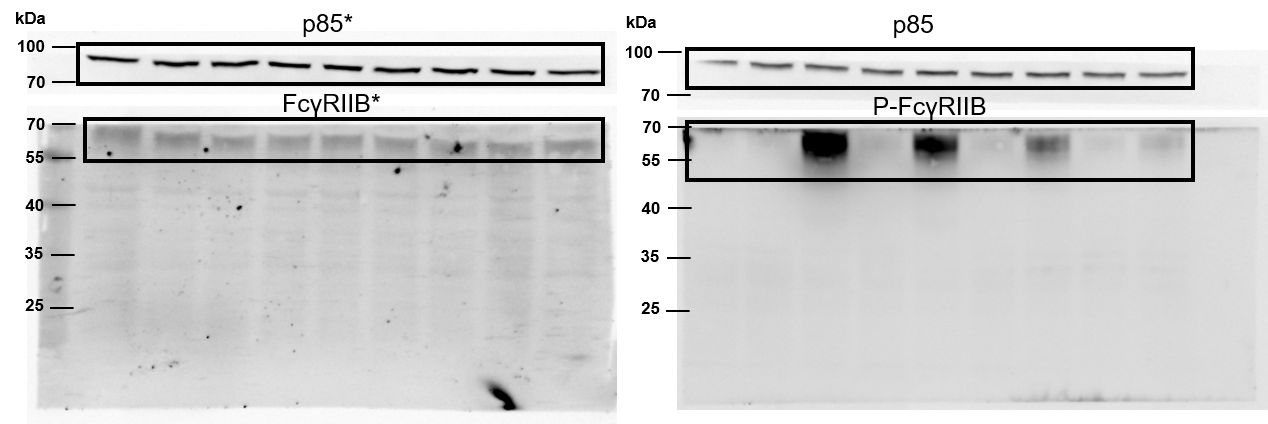


**Supplementary Figure S13:** Original blots belonging to bands shown in figure 2B.


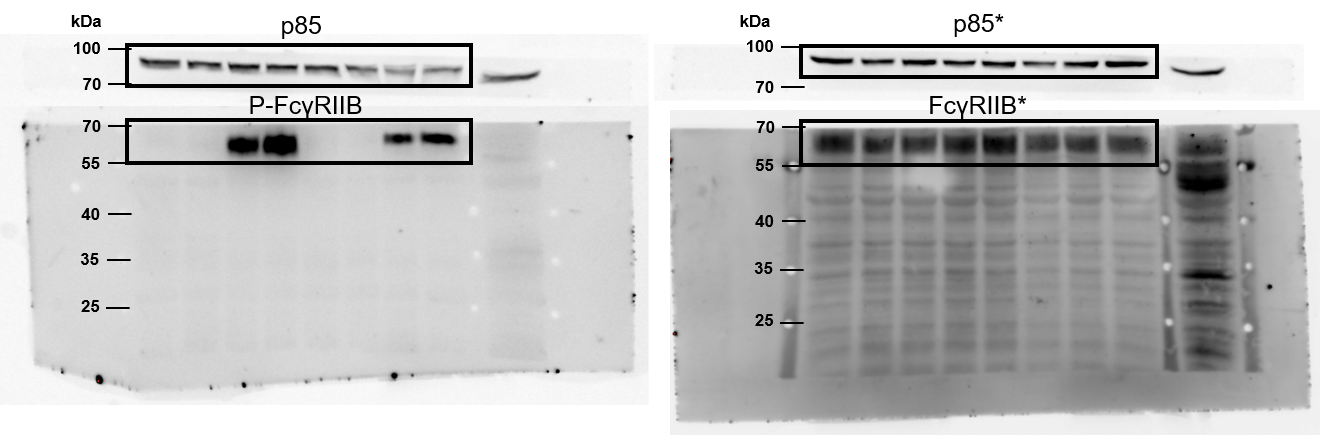


**Supplementary Figure S14:** Original blots belonging to bands shown in figure 2E.


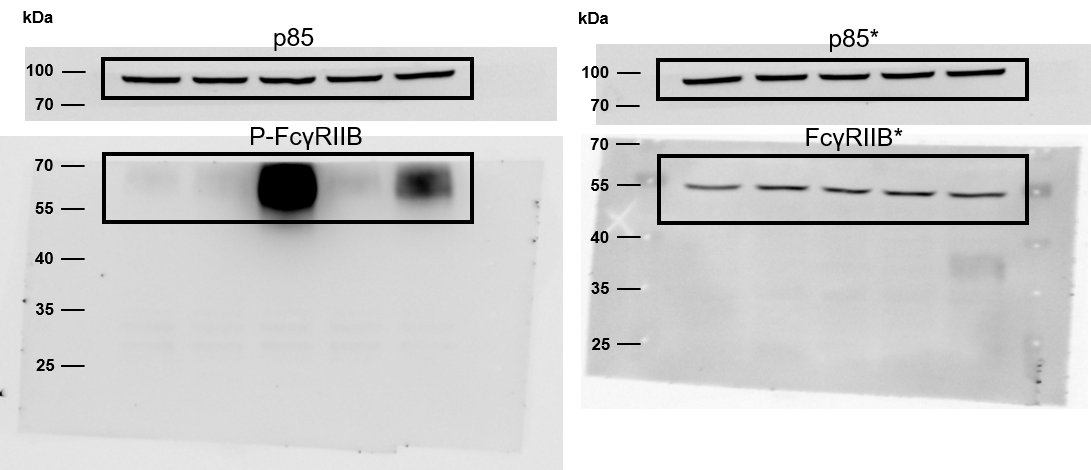


**Supplementary Figure S15:** Original blots belonging to bands shown in figure 2F.


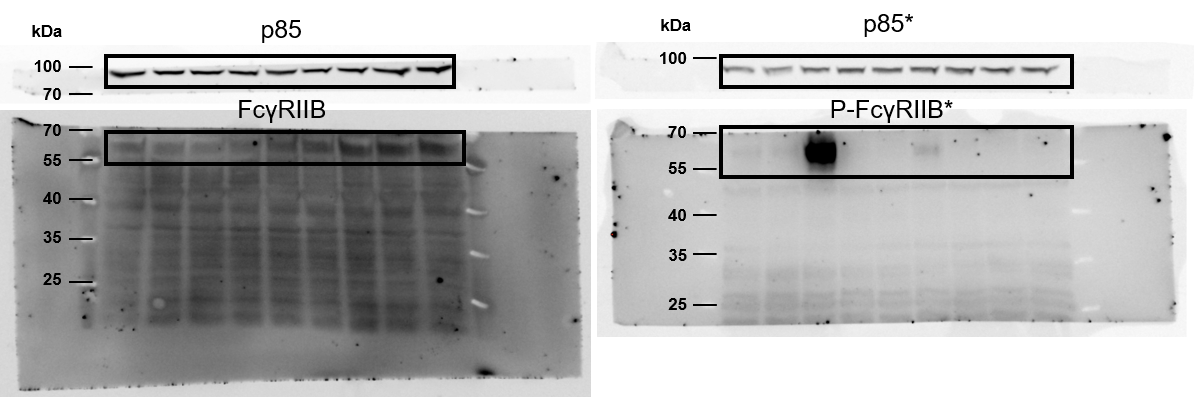


**Supplementary Figure S16:** Original blots belonging to bands shown in figure 3A.


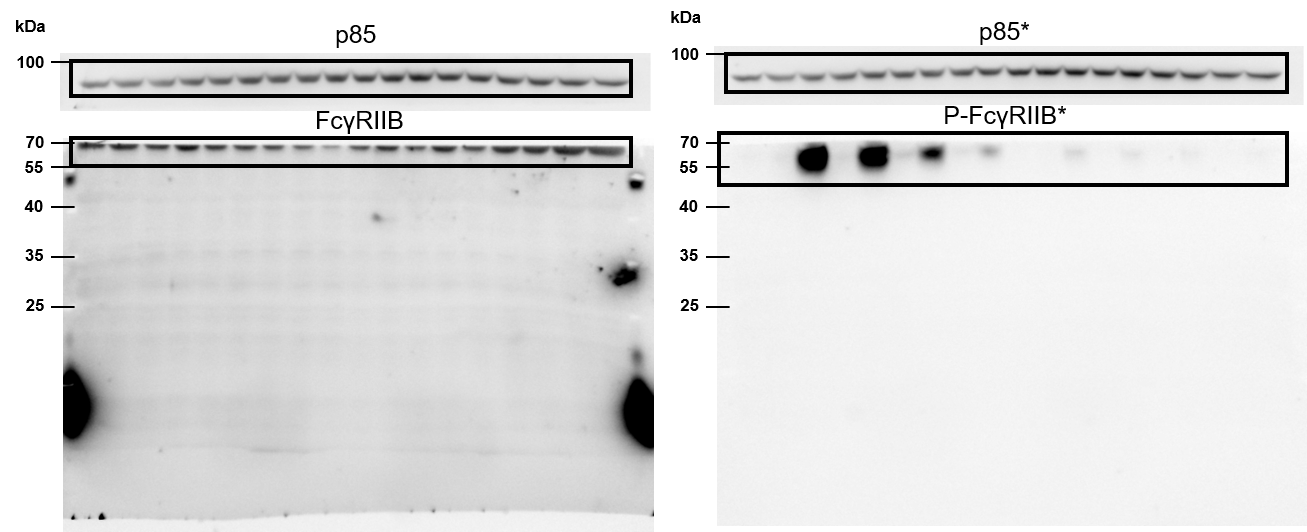


**Supplementary Figure S17:** Original blots belonging to bands shown in figure 3B.


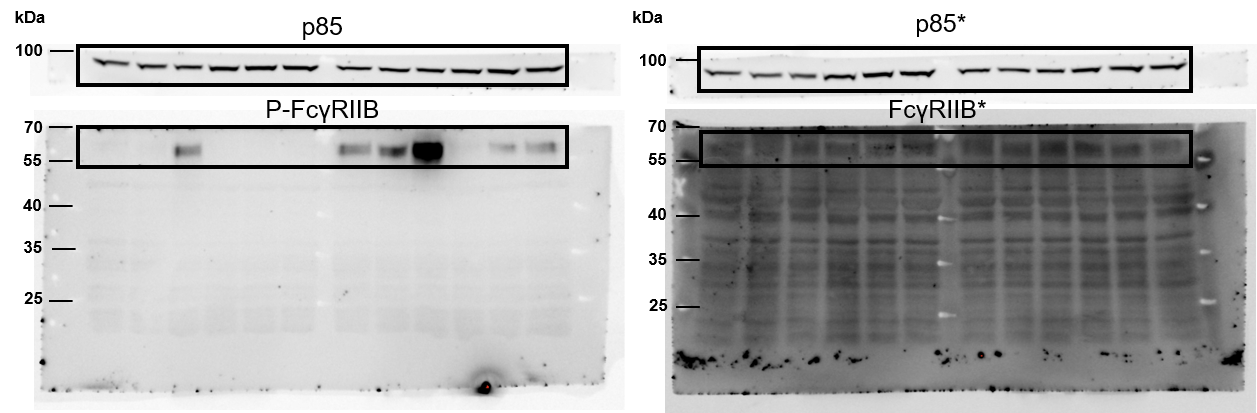


**Supplementary Figure S18:** Original blots belonging to bands shown in figure 3C.


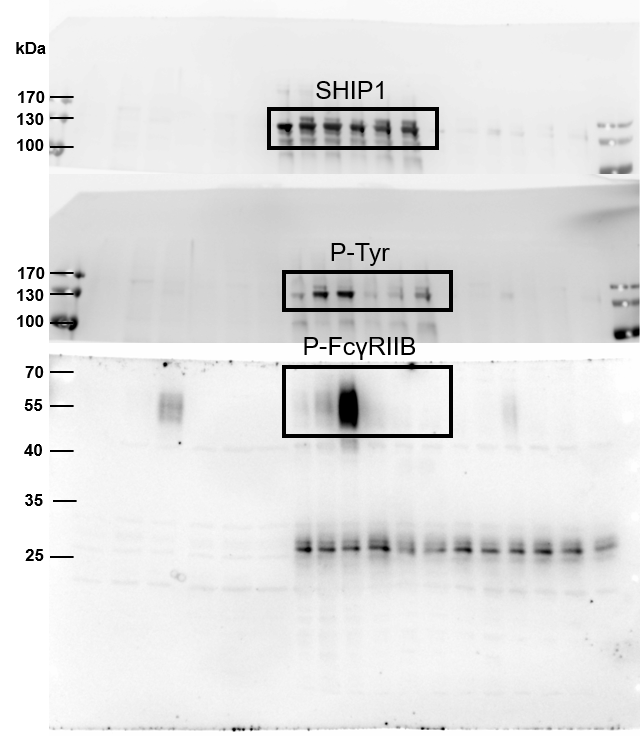


**Supplementary Figure S19:** Original blots belonging to bands shown in figure 3D.


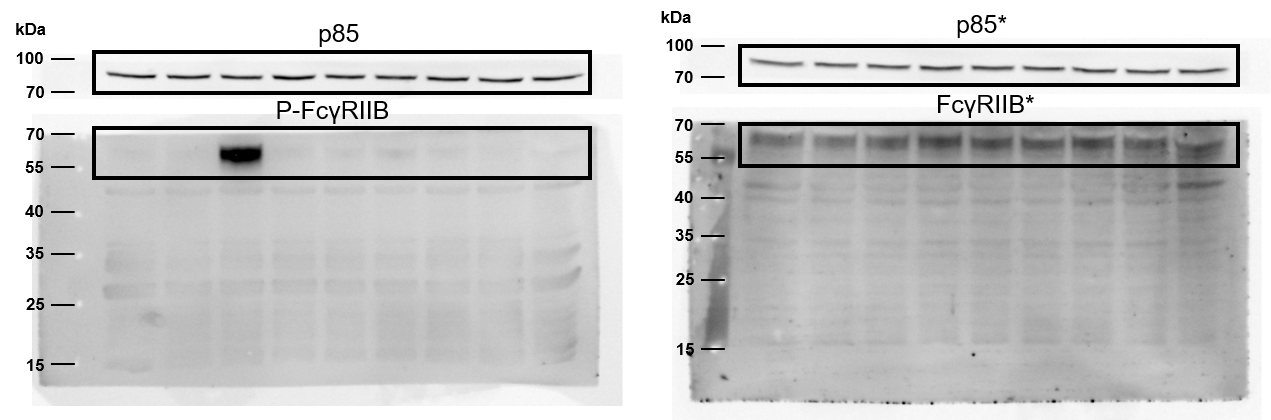


**Supplementary Figure S20:** Original blots belonging to bands shown in figure 5B.


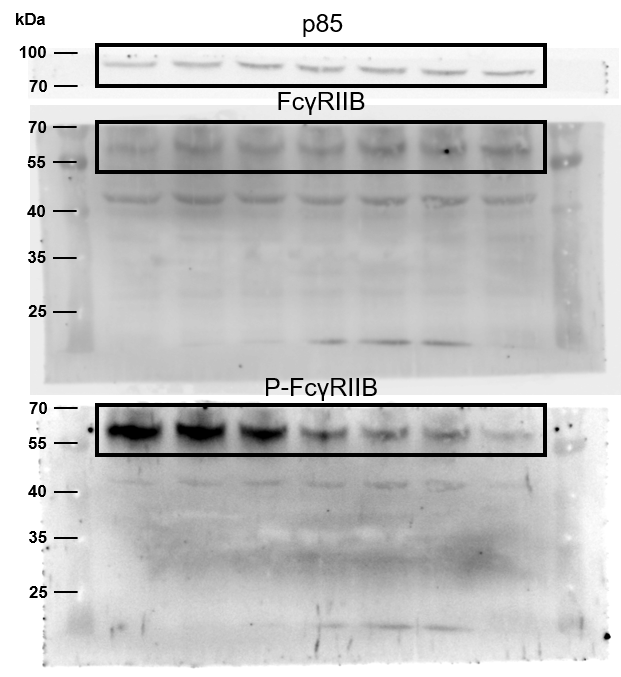


**Supplementary Figure S21:** Original blots belonging to bands shown in figure 5C.


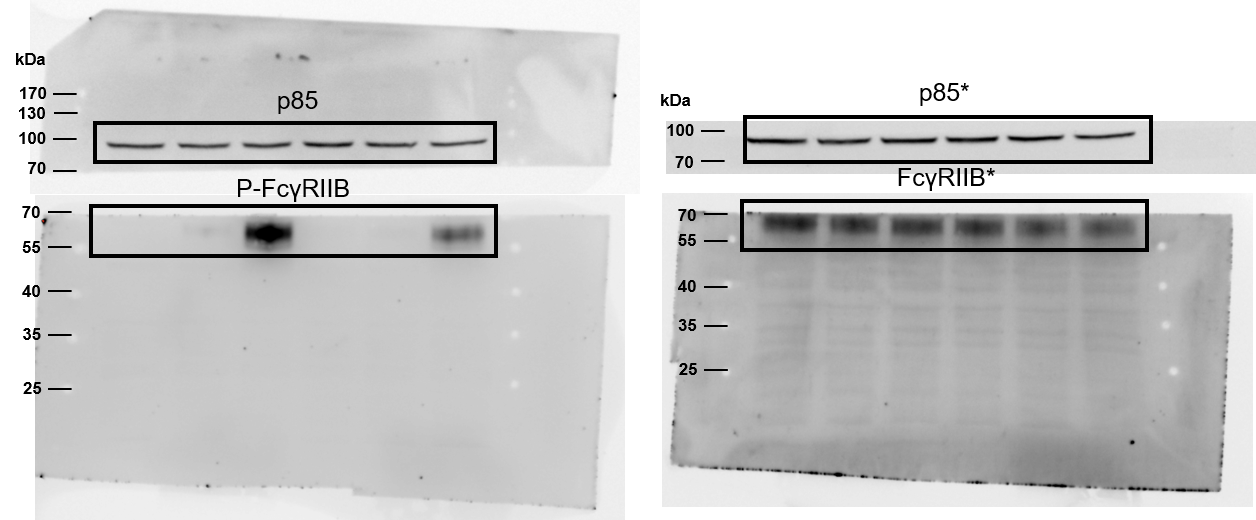


**Supplementary Figure S22:** Original blots belonging to bands shown in figure 5D.

# References:

1. Poplutz M, Levikova M, Lüscher-Firzlaff J, Lesina M, Algül H, Lüscher B, et al. Endotoxin tolerance in mast cells, its consequences for IgE-mediated signalling, and the effects of BCL3 deficiency. Sci Rep. 2017 Dec 3;7(1):4534. http://www.ncbi.nlm.nih.gov/pubmed/28674400
